# Bioactive Compounds from the Stems of *Clausena lansium*

**DOI:** 10.3390/molecules22122226

**Published:** 2017-12-14

**Authors:** Jie Liu, Chuang-Jun Li, Yi-Qian Du, Li Li, Hua Sun, Nai-Hong Chen, Dong-Ming Zhang

**Affiliations:** 1 State Key Laboratory of Bioactive Substance and Function of Natural Medicines, Institute of Materia Medica, Chinese Academy of Medical Sciences and Peking Union Medical College, Beijing 100050, China; znyspa.xf@163.com (J.L.); lichuangjun@imm.ac.cn (C.-J.L.); dyq08201105@163.com (Y.-Q.D.); annaleelin@imm.ac.cn (L.L.); sunhua@imm.ac.cn (H.S.); chennh@imm.ac.cn (N.-H.C.); 2 Beijing Research Institute of Chinese Medicine, Beijing University of Chinese Medicine, Beijing 100029, China

**Keywords:** *Clausena lansium*, phenolic glycosides, terpenoids, neuroprotective activities, hepatoprotective activities

## Abstract

In view of the significant neuroprotective effect of *Clausena lansium*, we continued to separate the *n*-butanol and the water extracts from the stems of *C. lansium* in order to find the leading compounds with significant activity. Two new phenolic glycosides, Clausenolside A–B (**1**–**2**), one new pair of phenolic enantiomers (**3a**, **3b**), and two new monoterpenoids, clausenapene A–B (**4**–**5**), together with twelve known analogues (**6**–**17**) were isolated from the stems of *C. lansium*. Compounds **1**–**17** were obtained from *C. lansium* for the first time. Compounds **3a**, **3b**, **4**, **16**, and **17** showed strong or moderate potential neuroprotective effects on inhibited PC12 cell injury induced by okadaic acid, and compound **9** exhibited strong potential hepatoprotective activities. Their structures were elucidated on the basis of spectroscopic analyses, including UV, IR, NMR experiments, and electronic circular dichroism (ECD) spectra.

## 1. Introduction

*Clausena lansium* (Lour.) Skeels (syn. *Clausena wampi* (Blanco) Oliv.; *Clausena punctate* (Sonn.) Rehd. & Wils.; *Cookia punctate* Sonn.; *Cookia wampi* Blanco; *Quinaria lansium* Lour.) is a minor member of the Rutaceae. It is an attractive shrub or small tree with somewhat grapelike fruit, similar to the citrus fruits and commonly called Wampee, False or Fool’s Curry [1]. It grows in the southern area of mainland China and is cultivated in Taiwan, Fujian, Guangdong, Guangxi, Hainan, etc. It also occurs in Vietnam, the Philippines, Malaysia, Singapore, Miami, etc. [2]. In traditional Chinese medicine, the leaves and roots of *C. lansium* were used to treat coughs, asthma, dermatological diseases, viral hepatitis, and gastro-intestinal diseases. The fruit were used to treat digestive disorders and the seeds were used to treat acute and chronic gastro-intestinal inflammation, ulcers, and so on [3].

**Figure 1:**
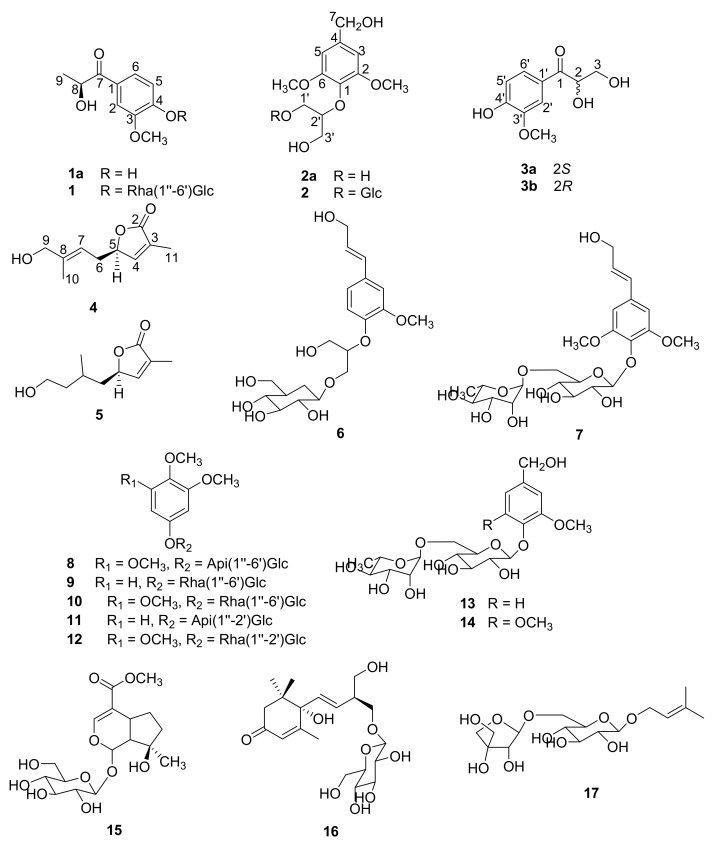
Structures of compounds **1**–**17**.

Various bioactive constituents including coumarins, carbazole alkaloids, and amide alkaloids have been isolated and identified from this plant [4,5,6]. Our research group has previously characterized a variety of new carbazole alkaloids, new amide glycosides, new coumarins, and new megastigmane glucoside from the leaves and stems of *C. lansium*, and several of these compounds showed selective neuroprotective and hepatoprotective effects [7,8,9,10,11,12]. However, the *n*-BuOH and the water extracts from the stems of *C. lansium* have not been investigated in detail. Herein, this paper reports on a further investigation of the water and *n*-BuOH extracts from the stems of *C. lansium,* which led to the isolation and characterization of two new phenolic glycosides (**1**–**2**), one new pair of phenolic enantiomers (**3a** and **3b**), two new monoterpenoids (**4**–**5**), together with twelve known analogues (**6**–**17**) (Figure 1). They were obtained from *C. lansium* for the first time. The determination of their absolute configurations occurred through spectroscopic analysis and electronic circular dichroism (ECD) experiments. Moreover, compounds **1**–**4** and **6**–**17** were assayed for their in vitro hepatoprotective and neuroprotective effects.

## 2. Results and Discussion

### 2.1. Purification and Characterization

**Figure 2:**
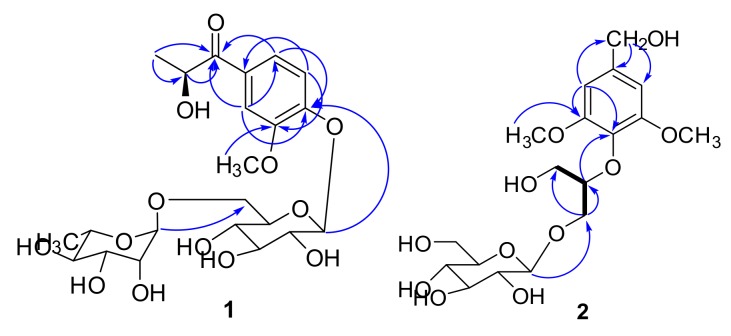
Key HMBC correlations of compounds **1** and **2**.

**Figure 3:**
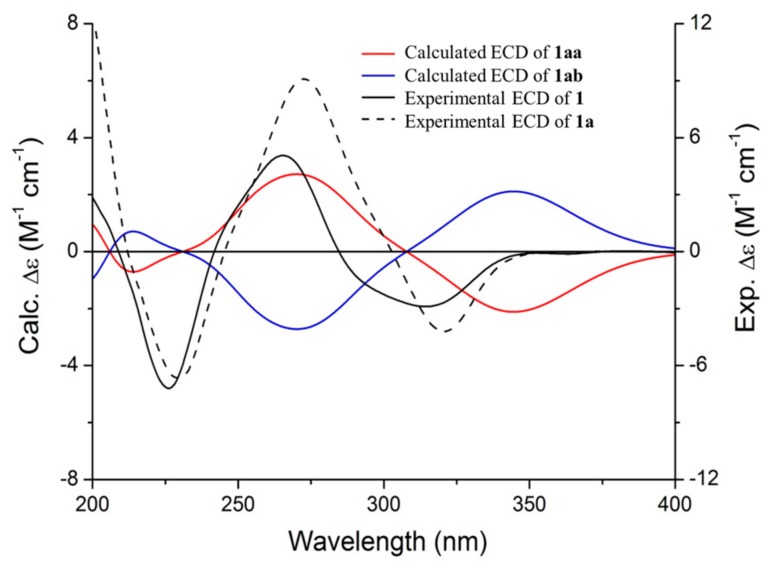
Calculated electronic circular dichroism (ECD) spectra of (8*S*) **1a** and (8*R*) **1a**-isomers and the experimental ECD of **1a** and **1**.

Clausenolside A (**1**) was obtained as a white, amorphous solid. Its molecular formula was deduced as C_22_H_32_O_13_ on the basis of its ^13^C-NMR and HRESIMS at *m*/*z* 527.1731 [M + Na]^+^, calculated as C_22_H_32_NaO_13_, 527.1735, implying seven indices of hydrogen deficiency. The ^1^H-NMR spectrum (Table 1) revealed three aromatic protons [δ_H_ 7.51 (1H, d, *J* = 2.0 Hz, H-2), 7.62 (1H, dd, *J* = 8.5, 2.0 Hz, H-6), 7.13 (1H, d, *J* = 8.5 Hz, H-5)], an oxygenated methine group δ_H_ 5.08 (1H, m, H-8), a methoxyl group δ_H_ 3.83 (3H, s, 3-OCH_3_), two methyl groups [δ_H_ 1.27 (3H, d, *J* = 6.7 Hz, H-9), 1.09 (3H, d, *J* = 6.2 Hz, H-6″)], and a set of protons for two glycosyl moieties, including two anomeric protons [δ_H_ 5.01 (1H, d, *J* = 6.1 Hz, H-1′), 4.52 (1H, br s, H-1″)]. The ^13^C-NMR and distortionless enhancement by polarization transfer (DEPT) spectra (Figure S3) along with the heteronuclear singular quantum correlation (HSQC) correlations (Figure S4) exhibited the presence of a benzene ring, a keto-carbonyl group, a methoxyl group, an oxygenated methine group, a methyl group, a glucosyl group, and a rhamnosyl group. On the basis of the NMR data analysis (Table 1), compound **1** was identified as a phenolic glycoside. In the heteronuclear multiple bond correlation (HMBC) spectrum (Figure S5), Correlations from H-8 to C-7 and from H-9 to C-7 and C-8 indicated that the oxygenated methine group was attached to C-7 and C-9. The correlations of H-2/C-4 (δ_C_ 150.6), C-6 (δ_C_ 122.7), C-7 (δ_C_ 200.3); H-6/C-7 (δ_C_ 200.3), C-4 (δ_C_ 150.6); H-5/C-1 (δ_C_ 128.5), C-3 (δ_C_ 148.7); and 3-OCH_3_/ C-3 (δ_C_ 148.7) demonstrated that the carbonyl was attached to C-1 and the methoxyl group was resonated at C-3. Correlations from H-1′ to C-4 and from H-1″ to C-6′ indicated that the rhamnosyl group was linked with C-6′ and the glucosyl group was linked with C-4 (Figure 2). The aglycone (**1a**) and sugar moieties were produced by acid hydrolysis of **1**. Sugar moieties were confirmed to be d-glucose and l-rhamnose by silylation followed with gas chromatography (GC) analysis. The absolute configuration of **1a** was defined as 8S by comparison of the experimental ECD spectra and the calculated ECD data using the time-dependent density functional theory (TDDFT) method at the B_3_LYP/6-31G (d) level [13]. The calculated ECD spectrum of (8S) **1a** (Figure 3) matched the experimental spectrum of **1a** and **1** very well, which indicated that the structure of **1a** had not changed in the process of acid hydrolysis and the absolute configuration of **1** was elucidated as 8S. Thus, the structure of **1** was assigned as depicted.

Clausenolside B (**2**) was obtained as an amorphous white powder. Its molecular formula was assigned as C_18_H_28_O_11_ based on the ^13^C-NMR spectroscopic data and HRESIMS (*m*/*z* 443.1532 [M + Na]^+^, calculated as C_18_H_28_NaO_11_ 443.1524), implying five indices of hydrogen deficiency. The NMR spectra (Table 1) of **2** were generally similar to those of compound **6 [14]**, except that the methoxyl group and the hydroxymethyl group of **2** replaced the hydrogen proton of C-6 and the propen-2-en-1-ol of C-4 replaced **6**. In the HMBC spectrum (Figure 2), the correlations from H-3 and H-5 to C-1 (δ_C_ 133.9), C-2 (δ_C_ 152.7), and C-7 (δ_C_ 63.0); from H-7 to C-3 (δ_C_ 103.5), C-4 (δ_C_ 138.2), and C-5 (δ_C_ 103.5) showed that the hydroxymethyl group was linked to C-4; the correlations from H-1′ to C-1 (δ_C_ 133.9), C-2′ (δ_C_ 67.6); from H-2′ to C-1′ (δ_C_ 81.4), C-3′ (δ_C_ 60.1); from H-1″ to C-2′ (δ_C_ 67.6) indicated that the propanetriol group was linked to C-1 and the β-glucopyranosyl unit was linked to C-2′. The correlations from OCH_3_ to C-2 (δ_C_ 152.7), C-6 (δ_C_ 152.7) demonstrated that the methoxyl group had attached to C-2 and C-6. The aglycone (**2a**) and sugar moiety were produced by an acid hydrolysis of **2**. Sugar moiety was confirmed to be d-glucose by silylation followed with GC analysis. Hence, the structure of **2** was assigned as shown.

Compound **3** (**3a**/**3b**) was obtained as a white powder. Its molecular formula C_10_H_12_O_5_ was deduced from the HRESIMS (*m*/*z* 235.0573 [M + Na]^+^, calculated as C_10_H_12_NaO_5_, 235.0577) and the ^13^C-NMR spectroscopic data, corresponding with five indices of hydrogen deficiency. The IR spectrum displayed absorptions characteristic of amino (3394 cm^−1^), amide (1667 cm^−1^), and of aromatic ring (1591, 1517, and 1465 cm^−1^) groups. According to ^1^H and ^13^C-NMR (Table 2), the plane structure of **3** was the same as 2,3-dihydroxy-1-(4-hydroxy-3-methoxyphenyl)-propan-1-one [15].

**Figure 4:**
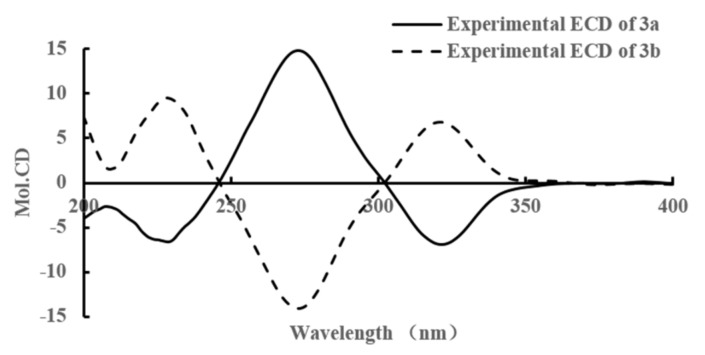
The circular dichroism (CD) Spectrum of compounds **3a** and **3b** in MeOH.

**Figure 5:**
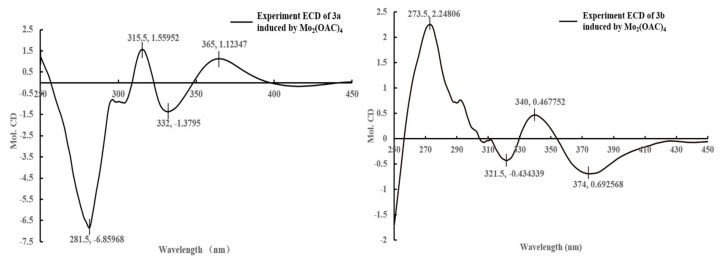
The CD spectrum of compounds **3a** and **3b** induced by Mo_2_(OAC)_4_ (dimolybdenum tetracetate) (the inherent CD of the diol was subtracted).

The specific rotation of **3** approached zero, and no Cotton effect was found in the ECD spectrum of **3**, indicating a racemic mixture. The subsequent chiral resolution of **3** afforded the anticipated enantiomers **3a** and **3b**, which showed mirror image-like ECD curves (Figure 4) and specific rotations {**3a**: ${\left[\alpha \right]}_{D}^{20}$ +3.6 (*c* 0.43, MeOH); **3b**: ${\left[\alpha \right]}_{D}^{20}$ −3.7 (*c* 0.66, MeOH)}. In order to confirm the absolute configuration of **3a** and **3b**, the 3′,4′-diol moiety of **3a** and **3b** was determined using induced circular dichroism (CD) spectra by Snatzke’s method [16,17]. A positive Cotton effect at 315 nm (Figure 5) in the induced CD spectrum indicated the 2R configuration for **3a** by means of the empirical helicity rule. Meanwhile, a negative Cotton effect at 321 nm (Figure 5) in the induced CD spectrum indicated the 2S configuration for **3b** by means of the empirical helicity rule. According to the above information, the absolute configuration of **3a** was 2R, and then the absolute configuration of **3b** was 2S. Therefore, compounds **3a** and **3b** were was assigned as (+)-(*R*)-2,3-dihydroxy-1-(4-hydroxy-3-methoxyphenyl)propan-1-one and (−)-(*S*)-2,3-dihydroxy-1-(4-hydroxy-3-methoxyphenyl)propan-1-one.

**Figure 6:**
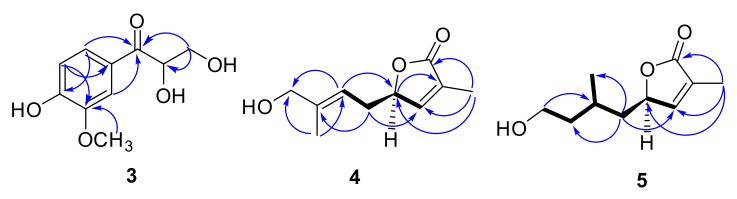
Key HMBC correlations of compounds **3**, **4** and **5**.

**Figure 7:**
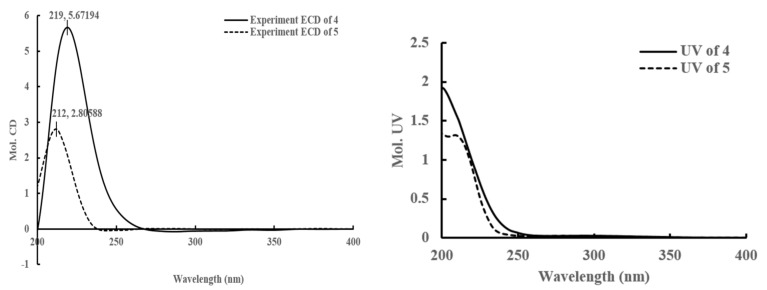
The CD and UV Spectrum of compounds **4** and **5** in MeOH.

Compound **4** was obtained as colorless oil. Its molecular formula was assigned as C_10_H_14_O_3_ based on the ^13^C-NMR spectroscopic data and the HRESIMS (*m*/*z* 205.0835 [M + Na]^+^, calculated as C_10_H_14_NaO_3_, 205.0835), implying four indices of hydrogen deficiency. The IR spectrum displayed characteristic absorptions of a five-membered ring unsaturated lactone group (1755 cm^−1^). The ^1^H-NMR (Table 3) spectrum showed a set of signals for two olefinic protons at δ_H_ 7.40 (1H, m, H-4), 5.37 (1H, m, H-7), one methine at δ_H_ 5.10 (1H, m, H-5), and two methyl groups at δ_H_ 1.64 (3H, s, H-10), δ_H_ 1.80 (3H, s, H-11). ^13^C-NMR (Table 3) and HSQC spectra (Figure S36) exhibited one carbonyl at δ_C_ 173.7, two double bonds at δ_C_ 128.1, 129.0, 130.8, 150.5, one oxymethine at δ_C_ 79.5, two methylenes at δ_C_ 42.7, 57.5, and two methyl groups at δ_C_ 16.4, 10.2. The ^1^H and ^13^C-NMR of **4** displayed signals characteristic of 3-substituted furanomonoterpene. The ^1^H, ^1^H- COSY (Figure 6) showed correlations between H-5 and H-4, H-6, as well as between H-8 and H-9. In the HMBC spectrum (Figure 6), the cross-peaks between H-4/C-2, C-3, C-5, C-11, H-5/C-3, C-7, C-8, H-9/C-6, C-7, and 11-CH_3_/C-2, C-4, C-5 were observed. A positive Cotton effect at 219 nm (Figure 7) in the CD spectrum indicated the 5R configuration for **4** by means of the octant rule of lactones [18,19]. According to above information, the plane structure of **4** was elucidated as (5*R*,*E*)-5-(4-hydroxy-3-methylbut-2-en-1-yl)-3-methylfuran-2(5*H*)-one and was given the trivial name clausenapene A.

Compound **5** was also obtained as a colorless oil. Its molecular formula was assigned as C_10_H_16_O_3_ based on the ^13^C-NMR spectroscopic data and the HRESIMS (*m*/*z* 207.0993 [M + Na]^+^, calculated as C_10_H_16_NaO_3_, 207.0992), implying three indices of hydrogen deficiency. The IR spectrum displayed absorptions that are characteristic of a five-membered ring unsaturated lactone group (1750 cm^−1^). A comparison of the ^1^H and the ^13^C-NMR (Table 3) of **5** with **4** suggested that their structures are closely similar, except, that the prenyl at C-5 of **4** were replaced by the isopentyl of **5**. The ^1^H and the ^1^H-COSY showed correlations (Figure 6) between H-5 and H-4, H-6; between H-6 and H-5, H-7; between H-7 and H-6, H-8, H-10; as well as between H-9 and H-8. In the HMBC spectrum, the cross-peaks between H-4/C-2, C-3, C-5, C-11, H-6/C-4, C-8, C-10, H-9/C-7, and 11-CH_3_/C-2, C-4, C-5 were observed. Compound **5** also exhibited a positive Cotton effect at 212 nm (Figure 7) in the CD spectrum, which indicated that the absolute configuration for **5** was R, as well as **4** by means of the octant rule of lactones [18,19]. According to the above information, the plane structure of **5** was elucidated as (5*R*)-5-(4-hydroxy-2-methylbutyl)-3-methylfuran-2(5*H*)-one and was given the trivial name clausenapene B.

### 2.2. Structure Identification of the Known Compounds

The nine known phenolic glycosides were identified as -*O*-β-d-glucopyranosyl-2-{2-methoxy-4-[1-(*E*)-propen-2-ol] phenoxyl}-ropane-3-ol (**6**) [14], 2,6-dimethoxy-4-(3-hydroxy-propen-1-yl)phenyl-4-*O*-α-l-rhamnopyranosyl-(1″-6′)-β-d-glucopyranoside (**7**) [20], 3,4,5-trimethoxyphenol 1-*O*-β-d-apiofuranosyl-(1″-6′)-β-d-glucopyranoside (**8**) [21], 3,4-dimethoxyphenyl-6-*O*-α-l-rhamnopyranosyl-(1″-6′)-β-d-glucopyranoside (**9**) [22], 12-*O*-(α-l-rhamnopyranosyl-(1″-6′)-β-d-glucopyranosyloxy)-3,4,5-trimethoxybenzene (**10**) [23], 3,4-dimethoxyphenol-*O*-β-d-apiofuranosyl-(1″-2′)-β-d-glucopyranoside (**11**) [24], khaephuoside A (**12**) [25], rhyncoside D (**13**) [26], 2-methoxy-4-hydroxymethylphenol-1-*O*-α-l-rhamnopyranosyl-(1″-6′)-β-d-glucopy-ranoside (**14**) [27]. Three terpene glycosides were identified as mussaenoside (**15**) [28], canangaionooside (**16**) [29], and isopentenol 1-*O*-β-d-apiofuranosyl-(1″-6′)-β-d-glucopyranoside (**17**) [30] on the basis of the analysis and the comparison of their spectroscopic data with literature values.

### 2.3. Neuroprotective Effect and Hepatoprotective Effect of Compounds ***1***–***4*** and ***6***–***17***

**Figure 8:**
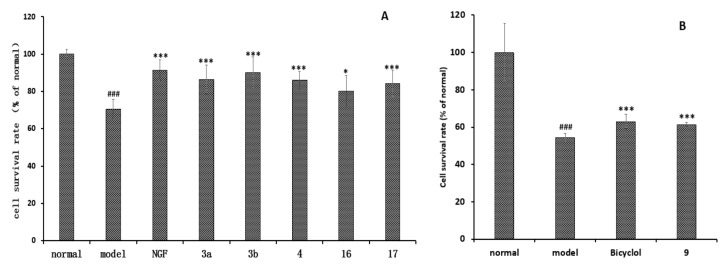
The neuroprotective and hepatoprotective effects of compounds isolated from *C. lansium*. (**A**) Neuroprotective effects of compounds **3a**, **3b**, **4**, **16**, **17** against okadaic acid-induced injury in PC12 Cells (10 μM, means ± SD, *n* = 6); (**B**) Hepatoprotective effects of compounds **1**–**4** and **6**–**17** (10 μM) against *N*-acetyl-*p*-aminophenol (APAP)-induced toxicity in HepG2 (human hepatocellular liver carcinoma cell line) cells (10 μM, means ± SD, *n* = 6). (^###^ *p* < 0.001 vs. normal, *** *p* < 0.001, * *p* < 0.1 vs. model).

Compounds **1**–**4** and **6**–**17** were evaluated for their neuroprotective effect on PC12 cells induced by okadaic acid (OKA) in vitro using the MTT method. As shown in Figure 8, at 10 μM, **3a**, **3b**, **4**, **16**, **17** increased the cell survival rate of the okadaic acid-treated group, while other compounds were inactive. Compounds **1**–**4** and **6**–**17** were also tested for hepatoprotective activities against *N*-acetyl-*p*-aminophenol (APAP)-induced toxicity in HepG2 (human hepatocellular liver carcinoma cell line) cells, using the hepatoprotective activity drug bicyclol as the positive control. As shown in Figure 8, compound **9** exhibited hepatoprotective activity, while other compounds were inactive.

### 2.4. Discussion

The Rutaceae have 150 genres and 1700 species and are distributed worldwide, although mainly in the tropics and subtropics. Their chemical composition mainly includes essential oils, alkaloids (phenylalanine anthranilic acid, carbazole, imidazolalkaloide, indolalkaloide, etc.), amides, cumarines (umbelliferonderivates, aesculetinderivates, daphnetinderivates, 5,7-dihydroxycumarins, isopropyldihydrofurocumarines, etc.), flavonoids, lignanes, phenolics, tetracyclic triterpenes and limonoids, diterpenes, pentacyclic triterpenes and saponins, etc. [31].

*Clausena lansium* (Lour.) Skeels, which belongs to the Rutaceae, has been cultivated in southern China and other warm areas of the world. Many chemical components, including carbazole alkaloids, coumarins, acyclic amides, cyclic amides, quinolones, phenyl glycosides, lactams and oxyneolignan were characterized from the stems, roots and leaves of *C. lansium*, and showed various biological activities. Some of the carbazole alkaloids, alkaloid glycosides, amides, and coumarins have exhibited potential anti-inflammatory activity, neuroprotective, hepatoprotective, and cytotoxicity activities [5,7,9,11,32,33,34,35,36]. However, as for the constituents from the fruit, seeds and peels, the references were very few. Few alkaloids, amides, and monoterpenes were isolated from the seeds of *C. lansium* [37,38]. The 8-hydroxypsoralen which showed antioxidant and cytotoxic activities, and the two new monoterpenoid coumarins (clauslactone V-W) which showed α-glucosidase inhibitory activity were obtained from the peels of *C. lansium* [6]. Some monoterpenoid coumarins and seven carbazole alkaloids were also isolated from the peels of *Clausena lansium* (Lour.) Skeels, and claulansine *J* exhibited moderate antibacterial activity against *Staphylococcus aureus* [39,40]. Three new jasmonoid glucosides, two new sesquiterpenes, two new coumarins, and others were isolated from the fruit of *C. lansium*. One coumarin was active against *S. aureus* and *S. dysenteriae,* and also exhibited moderate antioxidant activity, while one sesquiterpene, (+)-(*E*)-a-santalen-12-oic-acid, showed an inhibitory effect on *B. cereus* [41].

In this paper, the stems of *C. lansium* were collected in the Liuzhou commercial cultivation in Guangxi, China. Liuzhou, located in northern Guangxi, is a subtropical monsoon climate. Light, temperature, and water are very rich in Liuzhou. Therefore, the chemical components of *C. lansium* collected in Liuzhou could vary and be rich. Compounds **1**–**17** were obtained from the BuOH and the water extracts from the stems of *C. lansium* for the first time. The results are basically the same as those reported in the genus Rutaceae and for *C. lansium*. Some A,D-*seco*-limonoids have been characterized from the stems of *Clausena emarginata* [42] and we think that the two new monoterpenoids, clausenapenes A and B, may be decomposition products from the limonoides. In our studies, compounds **1**–**4** and **6**–**17** were assayed for the hepatoprotective and neuroprotective effects in vitro, in order to discover potential lead compounds. Herein, compounds **3a**, **3b**, **4**, **16**, and **17** showed strong or moderate potential neuroprotective effects by inhibiting PC12 cell injury induced by okadaic acid, and compound **9** exhibited strong potential hepatoprotective activities. It indicated that it is worth studying the chemical compositions of the BuOH and the water extracts of the stems of *C. lansium* to find more lead compounds.

## 3. Materials and Methods 

### 3.1. General Experimental Procedures

Optical rotations were measured on a JASCO P2000 automatic digital polarimeter (Jasco Corporation, Tokyo, Japan). UV spectra were recorded on a JASCO V-650 spectrophotometer (Jasco Corporation, Tokyo, Japan), CD spectra were measured on a JASCO J-815 spectropolarimeter (Jasco Corporation, Tokyo, Japan). IR spectra were recorded on a Nicolet 5700 spectrometer (Thermo Nicolet Corporation, Madison, SD, USA) using an FT-IR microscope transmission method. NMR spectra were acquired with Bruker AVIIIHD 600 (Bruker Corporation, Karlsruhe, Germany), Varian 600 and 400 (Varian Medical Systems, Inc., Palo Alto, CA, USA) in DMSO-*d*_6_. HRESIMS spectra were collected on UHPLC-Q Tof-MS (Agilent Technologies, Santa Clara, CA, USA). The MPLC system was composed of two C-605 pumps (Büchi, Flawil, Swizerland), a C-635 UV detector (Büchi), a C-660 fraction collector (Büchi), and an Octadecylsilyl (ODS) column (450 mm × 60 mm, 50 μm, 400 g; YMC, London, UK). Semi-preparative HPLC was conducted using a Shimadzu LC-6AD instrument (Shimadzu, Kyoto, Japan) with an SPD-20A detector and a Daicel Chiralpak AD-H column (250 mm × 10 mm, 5 μm). Preparative HPLC was also performed on a Shimadzu LC-6AD instrument (Shimadzu, Kyoto, Japan) with an YMC-Pack ODS-A column (250 mm × 20 mm, 5 μm). Column chromatography (CC) was performed with silica gel (200–300 mesh, Qingdao Marine Chemical Inc., Qingdao, China), MCI Gel (CHP20/P120, Mitsubishi chemical, Tokyo, Japan), SF-PRP 512A (100–200 mesh, Beijing Sunflower and Technology Development Co., Beijing, China), ODS (50 μm, YMC, Japan), and Sephadex LH-20 (GE, Upsala, Sweden). The TLC analysis was carried out on glass precoated silica gel GF254 plates. Spots were visualized under UV light or by spraying with 10% sulfuric acid in EtOH, followed by heating.

### 3.2. Cell Lines, Chemicals and Biochemical

PC12 cells (adrenal gland; pheochromocytoma) were purchased from the American Type Culture Collection. Human HepG2 hepatoma cells were purchased from the Cell Culture Centre at the Institute of Basic Medical Sciences, Chinese Academy of Medical Sciences. Dimethyl Sulphoxide (DMSO), Bicyclol, Okadaic Acid, 3-(3,4-dimehylthiazol-2-yl)-2,5-diphenyl-tetrazolium bromide (MTT) were obtained from Sigma (St. Louis, MO, USA). Dulbecco’s Modified Eagle’s Medium (DMEM), fetal bovine serum (FBS), and equine serum were purchased from Gibco BRL (New York, NY, USA). All other chemicals were of analytical grade and were commercially available.

### 3.3. Plant Materials

The stems of *C. lansium* were collected in Liuzhou, Guangxi, China, in March 2013, and were from commercial cultivation. *C. lansium* was identified by Engineer Guangri Long, Forestry of Liuzhou. A voucher specimen has been deposited at the Herbarium of Institute of Materia Medica, Chinese Academy of Medical Sciences and Peking Union Medical College (ID-S-2320). The 95% ethanol extract from the stems of *C. lansium* was stored in a refrigerator at −80 °C.

### 3.4. Extraction and Isolation

Air-dried, powdered stems of *C. lansium* (200 kg) were extracted with 95% ethanol (1000 L × 2 h × 3). The residue was suspended in water and then partitioned with EtOAc (3 × 40 L), and *n*-BuOH (3 × 40 L), successively.

After removing the solvent, the *n*-BuOH-soluble portion (850 g) was fractionated via a macroporous adsorbent resin (HPD-100) column with H_2_O, 30% EtOH, 60% EtOH, and 95% EtOH to yield four corresponding fractions A–D. Fraction B (420 g) was fractionated via silica gel column chromatography, eluting with CHCl_3_–MeOH–H_2_O (9:1:0.1, 8:2.5:0.3, 7:3:0.5, 6:4:0.4) to afford fifteen fractions B_1_–B_15_ on the basis of TLC analysis. Fraction B_1_ (39.6 g) was further separated by PRP-512A, silica gel column chromatography and preparative HPLC (detection at 210 nm, 60% CH_3_OH, 8 mL/min) to yield **4** (30 mg) and **5** (2 mg).

The water-soluble portion (25 L) was fractionated via a macroporous absorbent resin (HPD-100) column with H_2_O, 20% EtOH, 40% EtOH, and 95% EtOH to yield four corresponding fractions a–d. Fraction b (296 g) was further separated by silica gel column chromatography with CHCl_3_–MeOH–H_2_O (7:3:0.5, 6:4:0.4) to afford eight fractions B_1_–B_8_ on the basis of TLC analysis. Fraction B_1_ (10 g) was further separated by silica gel column chromatography, Sephadex LH-20, preparative HPLC (detection at 210 nm, 20% CH_3_CN, 8 mL/min) to yield 3 (29 mg). Compound 3 was further separated by semipreparative chiral HPLC (*n*-hexane–2-propanol, 2:1, 3 mL/min) to give 3a (2.8 mg) and 3b (2.1 mg). Fraction B_2_ (40 g) was further separated by MCI gel, and preparative HPLC (detection at 210 nm, 12% CH_3_CN, 8 mL/min) to yield 15 (29 mg). Fraction B_3_ (39 g) was further separated by PRP-512A with 10% EtOH, 15% EtOH, 20% EtOH, 30% EtOH, and 95% EtOH to yield fourteen fractions B_3-1_–B_3-14_. Fractions B_3-2_ was further separated by Sephadex LH-20, silica gel column chromatography, and preparative HPLC to yield 1 (18 mg), 2 (6 mg), 9 (4 mg), 13 (13 mg), and 14 (29 mg). Fraction B_3-7_ was further separated by Sephadex LH-20 and preparative HPLC to yield 6 (15 mg), 7 (11 mg), 8 (14 mg), 10 (118 mg), 11 (15 mg), 12 (6 mg), 16 (8 mg), 17 (17 mg).

### 3.5. Characterization

Compound **1**: white powder; ${\left[\alpha \right]}_{D}^{20}$ +61.9 (*c* 1.0 MeOH); UV (MeOH) λ_max_ (log ε) 206.4 (4.08), 223.6 (4.04), 269.6 (3.86), 304 (3.63) nm; IR (microscope) ν_max_ 3389, 2921, 1677, 1592, 1511, 1455, 1419, 1268, 1119, 1069 cm^−1^; ^1^H-NMR (DMSO-*d*_6_, 400 MHz) and ^13^C-NMR (DMSO-*d*_6_, 100 MHz), see Table 1; HRESIMS *m*/*z* 527.1731 [M + Na]^+^ (calculated for C_22_H_32_NaO_13_, 527.1735).

Compound **2**: white powder; ${\left[\alpha \right]}_{D}^{20}$ −6.16 (*c* 0.37 MeOH); UV (MeOH) λ_max_ (log ε) 207.0 (4.37), 271.2 (3.05) nm; IR (microscope) ν_max_ 3390, 2921, 2849, 1646, 1595, 1504, 1465, 1422, 1124, 1039 cm^−1^; ^1^H-NMR (DMSO-*d*_6_, 400 MHz) and ^13^C-NMR (DMSO-*d*_6_, 100 MHz), see Table 1; HRESIMS *m*/*z* 443.1532 [M + Na]^+^ (calculated for C_18_H_28_NaO_11_, 443.1524).

Compound **3a**: white powder; ${\left[\alpha \right]}_{D}^{20}$ +3.6 (*c* 0.1 MeOH); UV (MeOH) λ_max_ (log ε) 206.0 (4.41), 230.4 (4.29), 278.8 (4.14), 306.4 (4.10) nm; IR (microscope) ν_max_ 3394, 2921, 2849, 1667, 1591, 1517, 1465, 1427, 1286, 1107, 779 cm^−1^; ^1^H-NMR (DMSO-*d*_6_, 400 MHz) and ^13^C-NMR (DMSO-*d*_6_, 100 MHz), see Table 2; HRESIMS *m*/*z* 235.0573 [M + Na]^+^ (calculated for C_10_H_12_NaO_5_, 235.0577).

Compound **3b**: white powder; ${\left[\alpha \right]}_{D}^{20}$ −3.7 (*c* 0.1 MeOH); UV (MeOH) λ_max_ (log ε) 206.2 (4.38), 230.6 (4.24), 278.4 (4.10), 306.0 (4.06) nm; IR (microscope) ν_max_ 3394, 2921, 2849, 1647, 1591, 1517, 1468, 1420, 1285, 1107, 779 cm^−1^; ^1^H-NMR (DMSO-*d*_6_, 400 MHz) and ^13^C-NMR (DMSO-*d*_6_, 100 MHz), see Table 2; HRESIMS *m*/*z* 235.0573 [M + Na]^+^ (calculated for C_10_H_12_NaO_5_, 235.0577).

Compound **4**: colourless oil; ${\left[\alpha \right]}_{D}^{20}$ −50.1 (*c* 0.1, MeOH); UV (MeOH) λ_max_ (log ε) 203.6 (4.39) nm; CD (*c* 0.33, MeOH) λ_max_ 219 (5.67) nm; IR (microscope) ν_max_ 3418, 2925, 1755, 1659, 1441, 1384, 1261, 1101, 998 cm^−1^; ^1^H-NMR (DMSO-*d*_6_, 400 MHz) and ^13^C-NMR (DMSO-*d*_6_, 100 MHz), see Table 3; HRESIMS *m*/*z* 205.0835 [M + Na]^+^ (calculated for C_10_H_14_NaO_3_, 205.0835).

Compound **5**: colourless oil; ${\left[\alpha \right]}_{D}^{20}$ −58.5 (*c* 0.1 MeOH); UV (MeOH) λ_max_ (log ε) 201.4 (3.76) nm; CD (*c* 0.5, MeOH) λ_max_ 212 (2.80) nm; IR (microscope) ν_max_ 3394, 2921, 1750, 1646, 1468, 1117, 998 cm^−1^; ^1^H-NMR (DMSO-*d*_6_, 600 MHz) and ^13^C-NMR (DMSO-*d*_6_, 150 MHz), see Table 3; HRESIMS *m*/*z* 207.0993 [M + Na]^+^ (calculated for C_10_H_16_NaO_3_, 207.0992).

### 3.6. Acid Hydrolysis and GC Analysis of Compounds ***1*** and ***2***

Compound **2** (2 mg) was dissolved in 2 mol HCl-H_2_O (2 mL) and was then heated to 90 °C for 15 h. The reaction mixture was extracted with EtOAc. The aqueous layer was evaporated under vacuum, diluted repeatedly with H_2_O, and evaporated in vacuo to furnish a neutral residue. The residue was dissolved in anhydrous pyridine (1 mL), to which 2 mg of l-cysteine methyl ester hydrochloride was added. The mixture was stirred at 60 °C for 2 h, and after evaporation in vacuo to create dryness, 0.2 mL of *N*-trimethylsilylimidazole was added. The mixture was kept at 60 °C for another 2 h.

The reaction mixture was partitioned between n-hexane and H_2_O (2 mL each), and then the *n*-hexane extract was analyzed by GC under the following conditions: capillary column, HP-5 (30 m × 0.25 mm, with a 0.25 μm film; Dikma, Beijing, China); detection, FID; detector temperature, 280 °C; injection temperature, 250 °C; initial temperature 200 °C, then raised to 280 at 5 °C/min, final temperature maintained for 10 min; carrier, N_2_ gas. From the acid hydrolysate of 2, d-glucofuranurono-6, 3-lactone was confirmed by comparison of the retention time of its derivative, with that of an authentic sugar derivatized in a similar way, which showed a retention time of 18.4 min. The constituent sugar of compound 1 was identified by the same method as 2. Retention times of authentic sample were detected at 18.4 min (d-glucose) and 14.7 min (l-rhamnose) for 1.

### 3.7. Hepatoprotective Activity Assay

Human HepG2 hepatoma cells were cultured in a DMEM medium supplemented with 10% fetal calf serum, 100 U/mL penicillin, and 100 μg/mL streptomycin at 37 °C in a humidified atmosphere of 5% CO_2_ + 95% air. The cells were then passaged by treatment with 0.25% trypsin in 0.02% EDTA. The MTT assay was used to assess the cytotoxicity of test samples. The cells were seeded in 96-well multiplates. After an overnight incubation at 37 °C with 5% CO_2_, 10 μM test samples and APAP (final concentration of 8 mM) were added into the wells and incubated for another 48 h. Then, 100 μL of 0.5 mg/mL MTT was added to each well after the withdrawal of the culture medium and they were incubated for an additional 4 h. The resulting formazan was dissolved in 150 μL of DMSO after aspiration of the culture medium. The plates were placed on a plate shaker for 30 min and read immediately at 570 nm using a microplate reader [43]. The cell inhibitory rate (%) was calculated by (A_sample_ − A_blank_)/(A_untreated_ − A_blank_) × 100. *p*-Values of <0.05, <0.01, and <0.001 were regarded as statistically significant.

### 3.8. Neuroprotective Activity Assays

Pheochromocytoma (PC12) cells were incubated in DMEM, supplied with 5% fetal bovine serum and 5% equine serum as a basic medium. PC12 cells in the logarithmic phase were cultured at a density of 5000 cells per well in a 96-well microtiter plate. After 24 h incubation, the medium of the model group was changed to DMEM or a basic medium with 50 nM OKA for 24 h. Test compounds dissolved in DMSO were added to each well for >1000-fold dilution in the model medium at the same time. Each sample was tested in triplicate. After the incubation at 37 °C in 5% CO_2_ for 24 h, 10 μL of MTT (5 mg/mL) was added to each well and they were incubated for another 4 h. Then, the liquid in the wells was removed. Test compounds (100 μL) were added to each well. The absorbance was recorded on a microplate reader (Bio-Rad model 550, California, USA) at a wavelength of 570 nm [44]. Analysis of variance (ANOVA) followed by the Newman–Keuls post hoc test were performed to assess the differences between the relevant control and each experimental group. The cell inhibitory rate (%) was calculated by (A_sample_ − A_blank_)/(A_untreated_ − A_blank_) × 100. *p*-Values of <0.05, <0.01, and <0.001 were regarded as statistically significant.

## 4. Conclusions

In summary, this work described the isolation and the structure identification of two new phenolic glycosides (**1**–**2**), one new pair of phenolic enantiomers (**3a**, **3b**), and two new monoterpenoids (**4**–**5**), together with twelve known analogues (**6**–**17**). They were obtained from the stems of *C. lansium* for the first time. In addition, compounds **3a**, **3b**, **4**, **16**, and **17** showed strong or moderate potential neuroprotective effects on inhibiting PC12 cell injury induced by okadaic acid, and compound **9** exhibited strong potential hepatoprotective activities. In traditional Chinese medicine, the leaves, fruit, seeds, and the roots of *C. lansium* were used as folk medicine for treating many kinds of diseases. It has been reported that the stems of *C. lansium* have a characteristic chemical composition including carbazole, amide, quinolone alkaloids, coumarins, and others, which have various biological activities such as neuroprotective, anti-inflammatory, hepatoprotective, and cyctoxicity capacities. Therefore, not only the roots and the leaves but also the stems are important medicinal materials, which indicate that the chemical compositions and the biological activities of the stems of *C. lansium* are worth studying in order to find other compounds with potential activity.

## Figures and Tables

**Table 1:** ^1^H and ^13^C-NMR Spectroscopic Data of Compounds **1**, **1a**, **2** and **2a** (δ in ppm, *J* in Hz).

	1		1a		2		2a	
Position	δ_H_ *^a^*	δ_C_ *^b^*	δ_H_ *^a^*	δ_C_ *^b^*	δ_H_ *^a^*	δ_C_ *^b^*	δ_H_ *^a^*	δ_C_ *^b^*
1		128.5 s		130,1 s		133.9 s		134.1 s
2	7.51, d (2.0)	111.7 d	7.43, d (2.0)	112.1 d		152.7 s		152.7 s
3		148.7 s		148.2 s	6.63, s	103.5 d	6.64, s	103.5 d
4		150.6 s		150.2 s		138.2 s		138.1 s
5	7.13, d (8.5)	114.4 d	6.79, d (8.5)	115.5 d	6.63, s	103.5 d	6.64, s	103.5 d
6	7.62, dd (8.5, 2.0)	122.7 d	7.51, dd (8.5, 2.0)	124.2 d		152.7 s		152.7 s
7		200.3 s		200.0 s	4.43, d (5.7)	63.0 t	4.43, d (5.8)	63.0 t
8	5.08, m	68.5 d	4.98, q (6.6)	68.5 d				
9	1.27, d (6.7)	21.2 q	1.23, d (6.6)	21.8 q				
3-OCH3	3.83, s	55.7 q	3.78, s	56.0 q				
2,6-OCH3					3.76, s	55.9 q	3.76, s	55.9 q
1′	5.01, d (6.1)	99.6 d			3.98, m	81.4 d	3.82, m	83.4 d
2′	4.02, m	73.1 d			3.88, m; 3.71, m	67.6 t	3.59, m; 3.52, m	59.9 t
3′	3.28, m	76.7 d			3.56, m; 3.65, m	60.1 t	3.59, m; 3.52, m	59.9 t
4′	3.01, m	69.9 d						
5′	3.52, m	75.6 d						
6′	3.84, m; 3.40, m	66.5 t						
1″	4.52, br s	100.7 d			4.17, d (7.7)	103.4 d		
2″	3.46, m	70.4 d			2.94, m	73.5 d		
3″	3.58, m	70.7 d			3.04, m	76.7 d		
4″	3.13, m	72.0 d			3.09, m	70.0 d		
5″	3.44, m	68.3 d			3.14, m	76.8 d		
6″	1.09, d (6.2)	17.9 q			3.42, m; 3.61, m	61.0 t		

*^a^* In DMSO-*d*_6_ (600 MHz), *^b^* In DMSO-*d*_6_ (150 MHz). Coupling constants (*J*) in Hz are given in parentheses. The assignments were based on HSQC and HMBC experiments.

**Table 2:** ^1^H and ^13^C-NMR Spectroscopic Data of Compounds **3** (δ in ppm, *J* in Hz).

	3	
Position	δ_H_ *^a^*	δ_C_ *^b^*
1		198.3 s
2	4.96, t (4.7)	73.9 d
3a	3.69, dd (11.3, 4.2)	64.5 t
3b	3.59, dd (11.3, 4.9)	
1′		126.9 s
2′	7.48, s	111.7 d
3′		147.5 s
4′		151.9 s
5′	6.87, d (8.1)	114.7 d
6′	7.56, d (8.1)	123.6 d
6-OCH_3_	3.82, s	55.6 q

*^a^* In DMSO-*d*_6_ (400 MHz), *^b^* In DMSO-*d*_6_ (100 MHz). Coupling constants (*J*) in Hz are given in parentheses. The assignments were based on HSQC and HMBC experiments.

**Table 3:** ^1^H-NMR and ^13^C-NMR Spectroscopic Data of Compounds **4** and **5** (δ in ppm, *J* in Hz).

	4		5	
Position	δ_H_ *^a^*	δ_C_ *^b^*	δ_H_ *^a^*	δ_C_ *^b^*
2		173.7 s		173.8 s
3		128.1 s		127.7 s
4	7.36, m	150.5 d	7.43, m	151.2 d
5	5.10, m	79.5 d	5.05, m	79.4 d
6	2.36, dd (5.2, 14.0); 2.19, dd (8.2, 14.0)	42.7 t	1.51, m; 1.42, m	40.4 t
7	5.37, m	129.0 d	1.77, m	26.4 d
8		130.8 s	1.47, m; 1.30, m	40.1 t
9	3.95, d (6.0)	57.5 t	3.40, m	58.5 t
10	1.64, s	16.4 q	0.93, d (7.8)	19.2 q
11	1.80, s	10.2 q	1.80, s	10.2 q

*^a^* In DMSO-*d*_6_ (600 MHz), *^b^* In DMSO-*d*_6_ (150 MHz). Coupling constants (*J*) in Hz are given in parentheses. The assignments were based on HSQC and HMBC experiments.

## Supplementary Materials

The following are available online.
