# Supplementary material for: Bioactive Compounds from the Stems of Clausena lansium
Source: Molecules. 2017 Dec 14;22(12):2226. doi: 10.3390/molecules22122226 (PMC6149816; doi:10.3390/molecules22122226)
Supplement: Supplementary file 1 [file molecules-22-02226-s001.pdf]

Supporting information

Bioactive Compounds from the stems of *Clausena lansium*

Jie Liu [1, 2], Chuang-Jun Li [1], Yi-Qian Du [1], Li Li [1], Hua Sun [1], Nai-Hong Chen [1] and Dong-Ming Zhang [1*]

[1] State Key Laboratory of Bioactive Substance and Function of Natural Medicines, Institute of Materia Medica, Chinese Academy of Medical Sciences and Peking Union Medical College, Beijing 100050, People's Republic of China; znyspa.xf@163.com (J.L.); lichuangjun@imm.ac.cn (C.-J.L.); dyq08201105@163.com (Y. –Q. D.); annaleelin@imm.ac.cn (L.L.); sunhua@imm.ac.cn (H.S.); chennh@imm.ac.cn (N.-H.C.);

[2] Beijing Research Institute of Chinese Medicine, Beijing University of Chinese Medicine, Beijing 100029, People's Republic of China;

* Correspondence: zhangdm@imm.ac.cn; Tel.: + 86-010-631-65227

Index

Figure S1. The ^{1}H NMR Spectrum of compound **1**（400 MHz，DMSO-d_6）........ 4

Figure S2. The ^{13}C NMR Spectrum of compound **1**（100 MHz，DMSO-d_6）........ 5

Figure S3. The DEPT Spectrum of compound **1**........ 6

Figure S4. The HSQC Spectrum of compound **1**........ 7

Figure S5. The HMBC Spectrum of compound **1**........ 8

Figure S6. The HRESIMS Spectrum of compound **1**........ 9

Figure S7. The IR Spectrum of compound **1**........ 10

Figure S8. The ^{1}H NMR Spectrum of compound **1a**（600 MHz，DMSO-d_6）........ 11

Figure S9. The ^{13}C NMR Spectrum of compound **1a**（150 MHz，DMSO-d_6）........ 12

Figure S10. Calculated ECD spectra of (8S)**1a** and (8R)**1a** and the experimental ECD of **1a** and **1**........ 13

Figure S11. GC chromatogram of sugar motieties silylationed after acid hydrolysis of **1**........ 14

Figure S12. The ^{1}H NMR Spectrum of compound **2**（400 MHz，DMSO-d_6）........ 15

Figure S13. The ^{13}C NMR Spectrum of compound **2**（100 MHz，DMSO-d_6）........ 16

Figure S14. The DEPT Spectrum of compound **2**........ 17

Figure S15. The HSQC Spectrum of compound **2**........ 18

Figure S16. The ^{1}H, ^{1}H-COSY Spectrum of compound **2**........ 19

Figure S17. The HMBC Spectrum of compound **2**........ 20

Figure S18. The HRESIMS Spectrum of compound **2**........ 21

Figure S19. The IR Spectrum of compound **2**........ 22

Figure S20. The ^{1}H NMR Spectrum of compound **2a**（600 MHz，DMSO-d_6）........ 23

Figure S21. The ^{13}C NMR Spectrum of compound **2a**（150 MHz，DMSO-d_6）........ 24

Figure S22. GC chromatogram of sugar motiety silylationed after acid hydrolysis of **2**........ 25

Figure S23. The ^{1}H NMR Spectrum of compound **3**（400 MHz，DMSO-d_6）........ 26

Figure S24. The ^{13}C NMR Spectrum of compound **3**（100 MHz，DMSO-d_6）........ 27

Figure S25. The HSQC Spectrum of compound **3**........ 28

Figure S26. The HMBC Spectrum of compound **3**........ 29

Figure S27. The HRESIMS Spectrum of compound **3**........ 30

Figure S28. The IR Spectrum of compound **3a**........ 31

Figure S29. The IR Spectrum of compound **3b**........ 32

Figure S30. The experimental CD Spectrum of compound **3a** and **3b** in MeOH........ 33

Figure S31. The $Mo_2(OAc)_4$ Induced CD Spectrum of compound **3a** 34
Figure S32. The $Mo_2(OAc)_4$ Induced CD Spectrum of compound **3b** 35
Figure S33. The ^{1}H NMR Spectrum of compound **4**（600 MHz，DMSO-d_6） 36
Figure S34. The ^{13}C NMR Spectrum of compound **4**（150 MHz，DMSO-d_6） 37
Figure S35. The DEPT Spectrum of compound **4** 38
Figure S36. The HSQC Spectrum of compound **4** 39
Figure S37. The ^{1}H, ^{1}H-COSY Spectrum of compound **4** 40
Figure S38. The HMBC Spectrum of compound **4** 41
Figure S39. The HRESIMS Spectrum of compound **4** 42
Figure S40. The IR Spectrum of compound **4** 43
Figure S41. The CD Spectrum of compound **4** 44
Figure S42. The ^{1}H NMR Spectrum of compound **5**（600 MHz，DMSO-d_6） 45
Figure S43. The ^{13}C NMR Spectrum of compound **5**（150 MHz，DMSO-d_6） 46
Figure S44. The DEPT Spectrum of compound **5** 47
Figure S45. The HSQC Spectrum of compound **5** 48
Figure S46. The ^{1}H, ^{1}H-COSY Spectrum of compound **5** 49
Figure S47. The HMBC Spectrum of compound **5** 50
Figure S48. The HRESIMS Spectrum of compound **5** 51
Figure S49. IR Spectrum of compound **5** 52
Figure S50. CD Spectrum of compound **5** 53

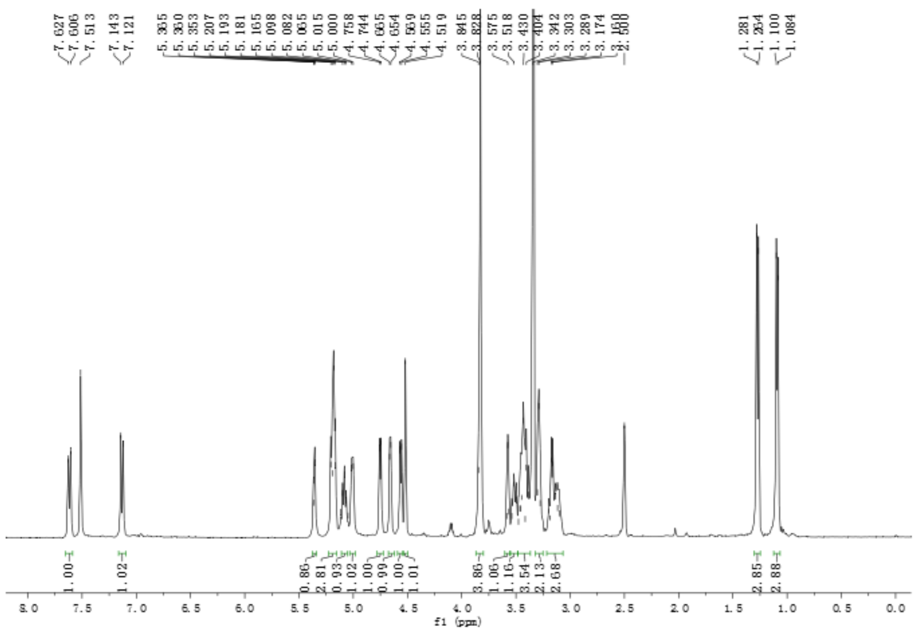

Figure S1. The ^{1}H NMR Spectrum of compound 1（400 MHz，DMSO-*d*6）

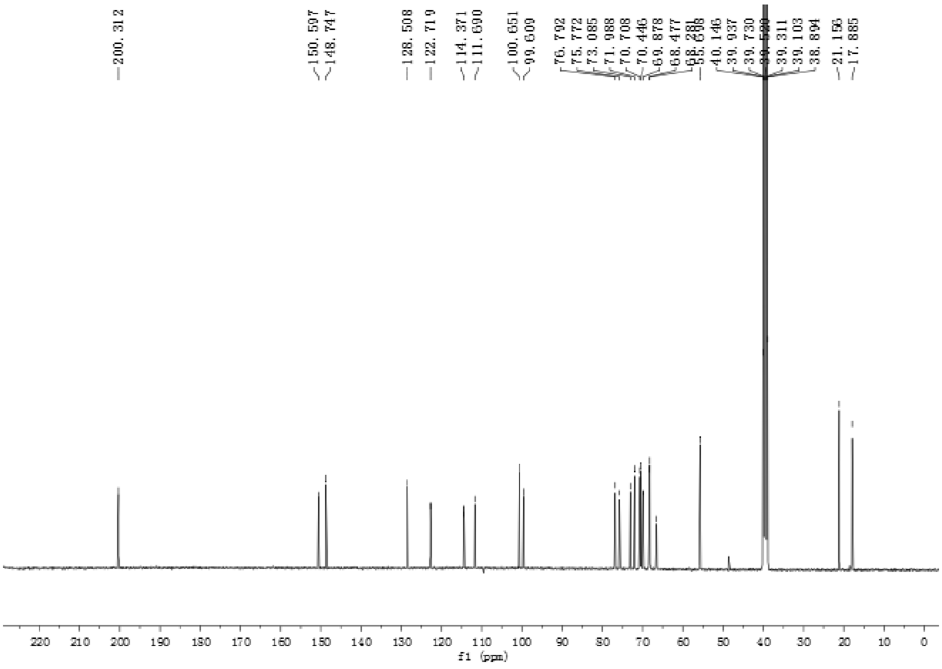

Figure S2. The ^{13}C NMR Spectrum of compound 1（100 MHz，DMSO-*d*$_6$）

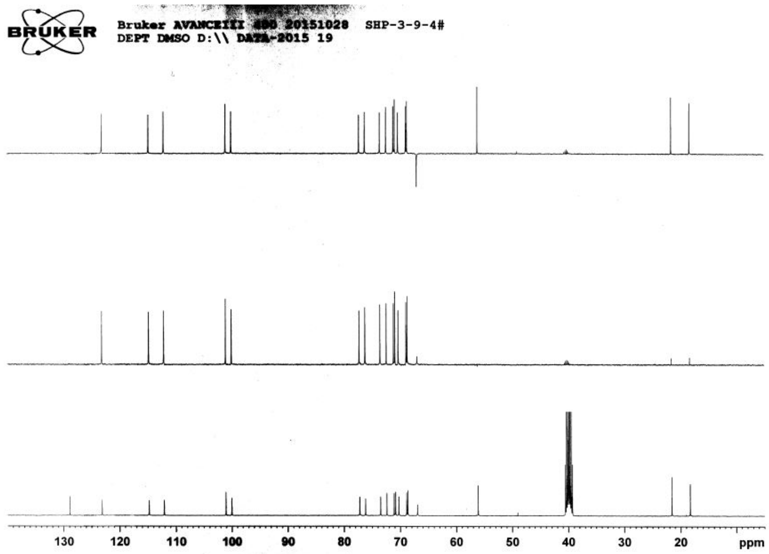

Figure S3. The DEPT Spectrum of compound 1

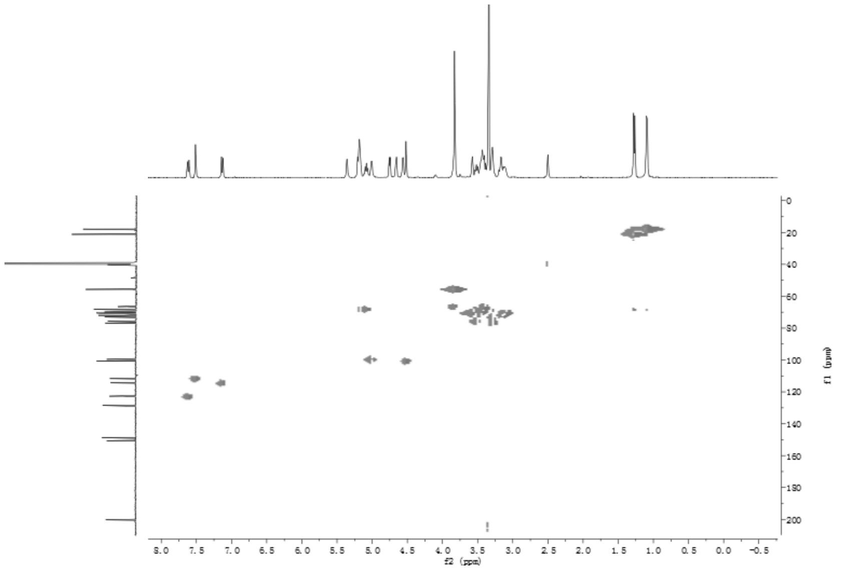

Figure S4. The HSQC Spectrum of compound 1

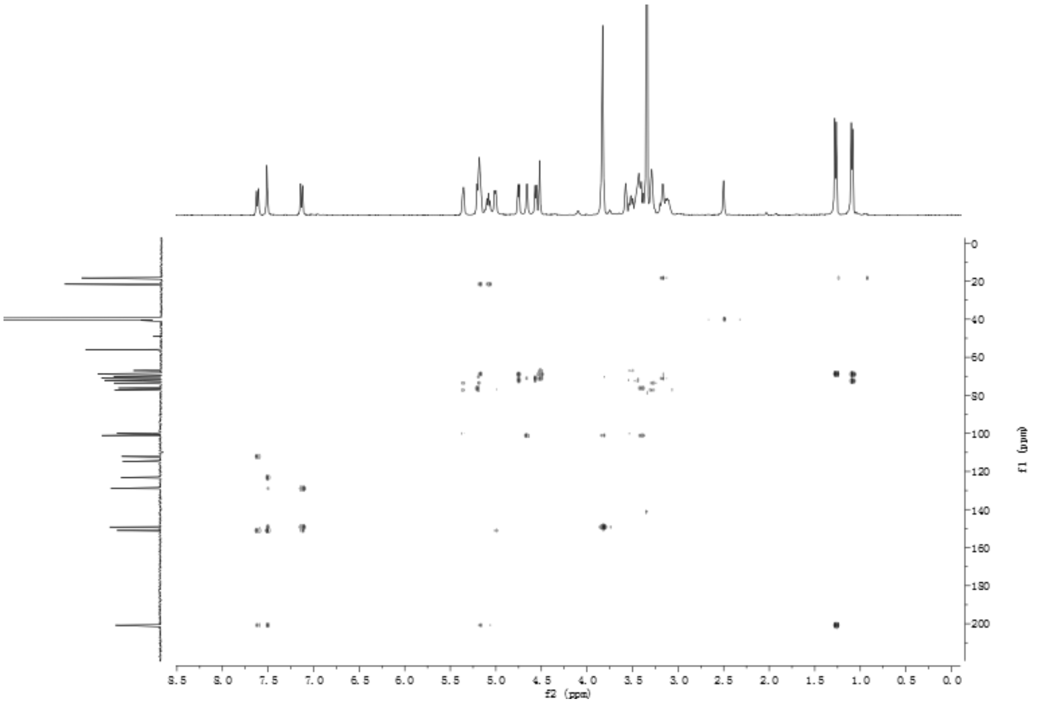

Figure S5. The HMBC Spectrum of compound 1

MS Formula Results: + Scan (3.658 min) Sub (2016031801.d)

m/z	Ion	Formula	Abundance
527.1731	(M+Na)+	C22 H32 Na O13	55974.3

Best	Formula (M)	Ion Formula	Score	Cross Sco	Mass	Calc Mass	Calc m/z	Diff (ppm)	Abs Diff (ppm)	Mass Match	Abund Match	Sp
✔	C22 H32 O13	C22 H32 Na O13	99.93		504.1839	504.1843	527.1735	0.82	0.82	99.98	99.93	
	C35 H24 N2 O2	C35 H24 N2 Na O2	96.5		504.1839	504.1838	527.173	-0.2	0.2	100	87.95	

Figure S6. The HRESIMS Spectrum of compound 1

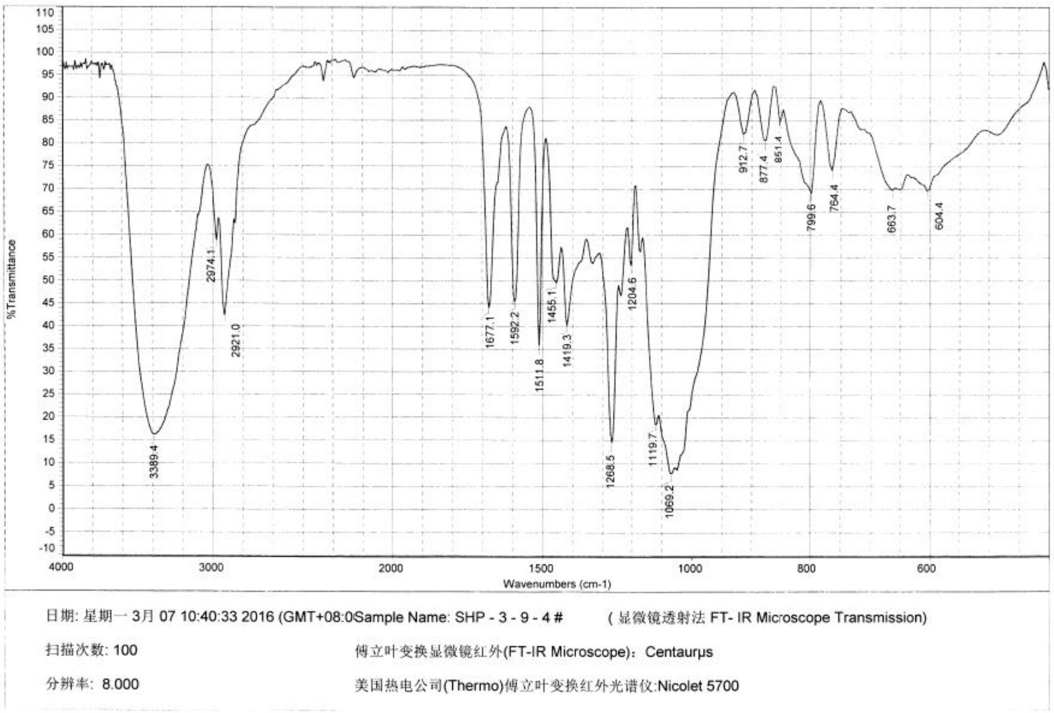

Figure S7. The IR Spectrum of compound 1

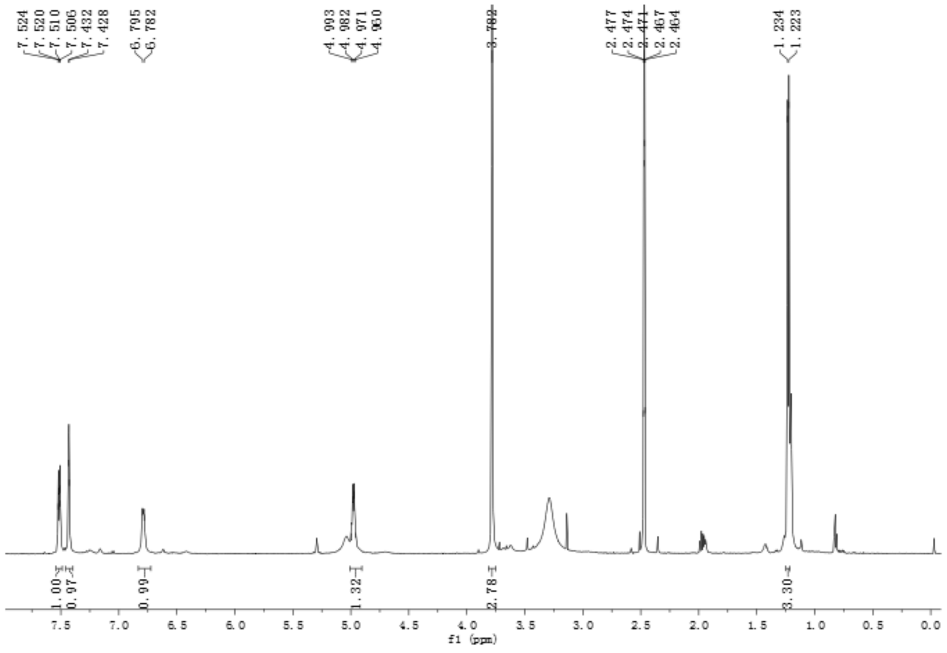

Figure S8. The ^{1}H NMR Spectrum of compound 1a（600 MHz，DMSO-*d*6）

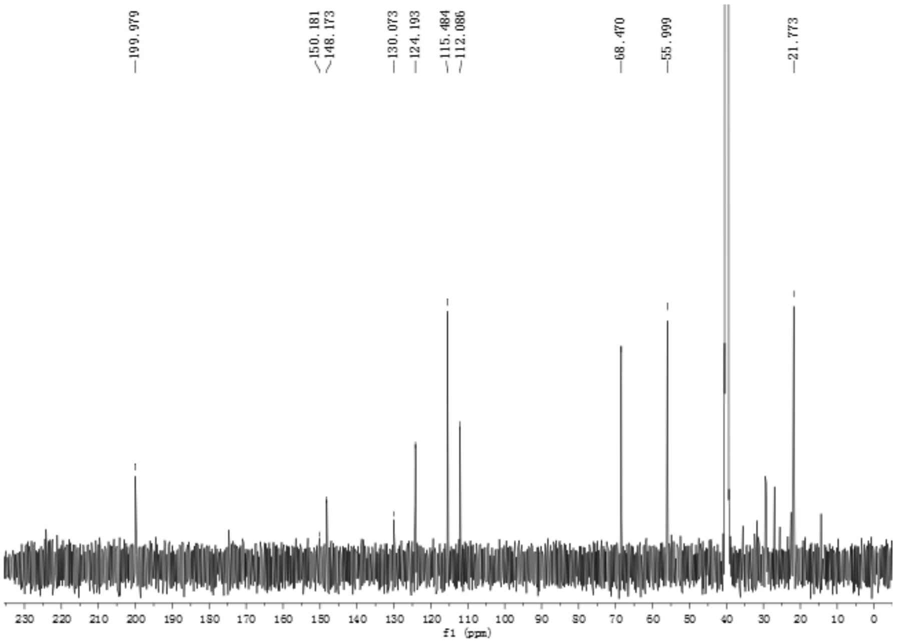

Figure S9. The ^{13}C NMR Spectrum of compound 1a（150 MHz，DMSO-d_6）

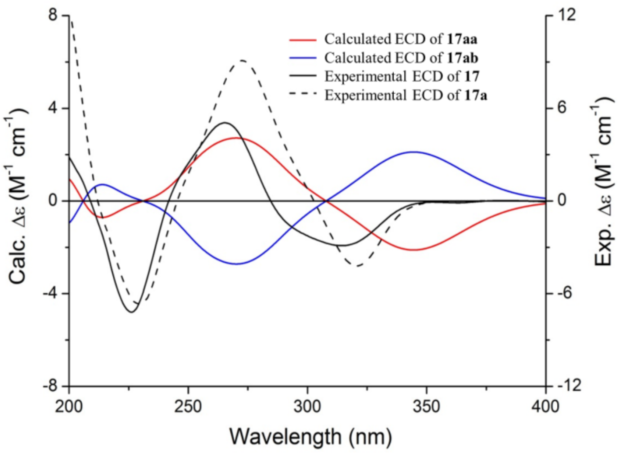

Figure S10. Calculated ECD spectra of (8*S*)1a and (8*R*)1a and the experimental ECD of 1a and 1

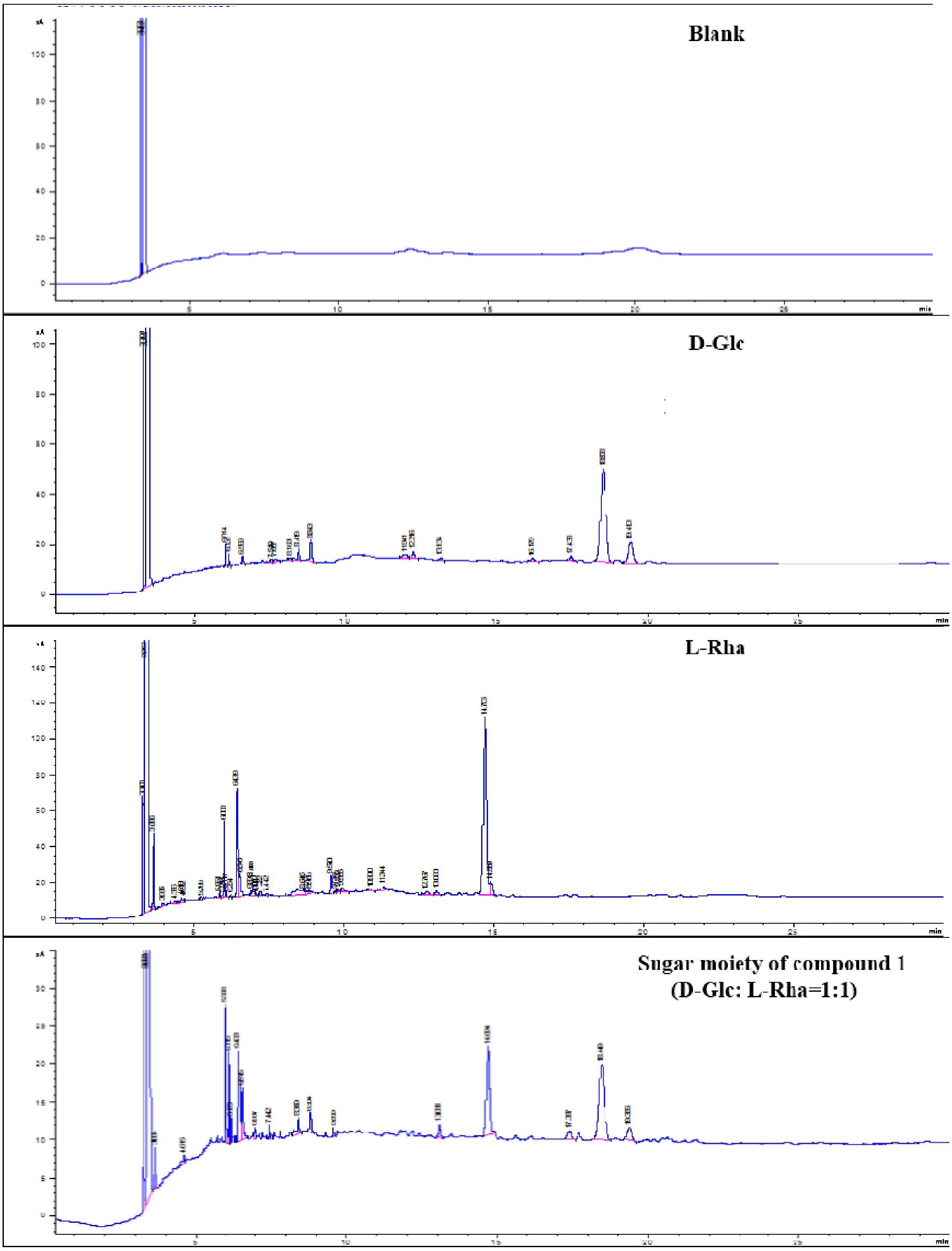

Figure S11. GC chromatogram of sugar motieties silylationed after acid hydrolysis of 1

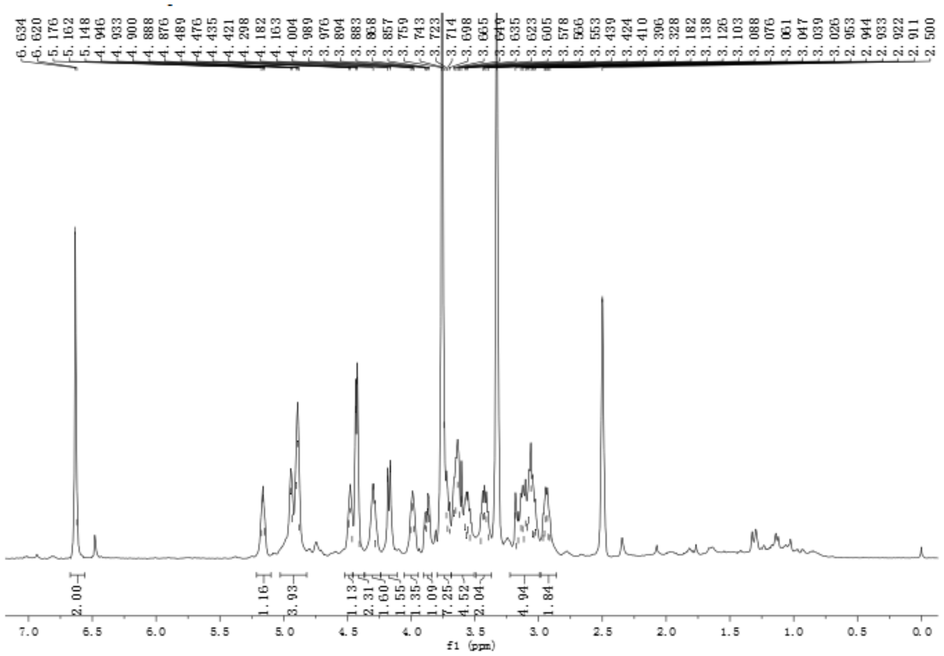

Figure S12. The ^{1}H NMR Spectrum of compound 2（400 MHz，DMSO-*d*6）

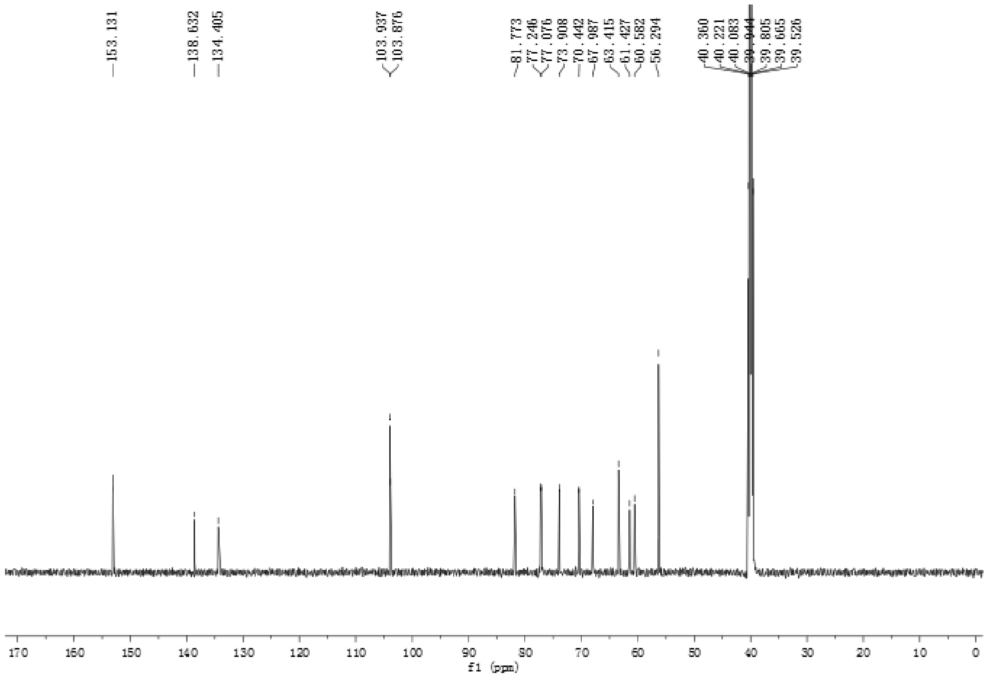

Figure S13. The ^{13}C NMR Spectrum of compound 2（100 MHz，DMSO-*d*$_6$）

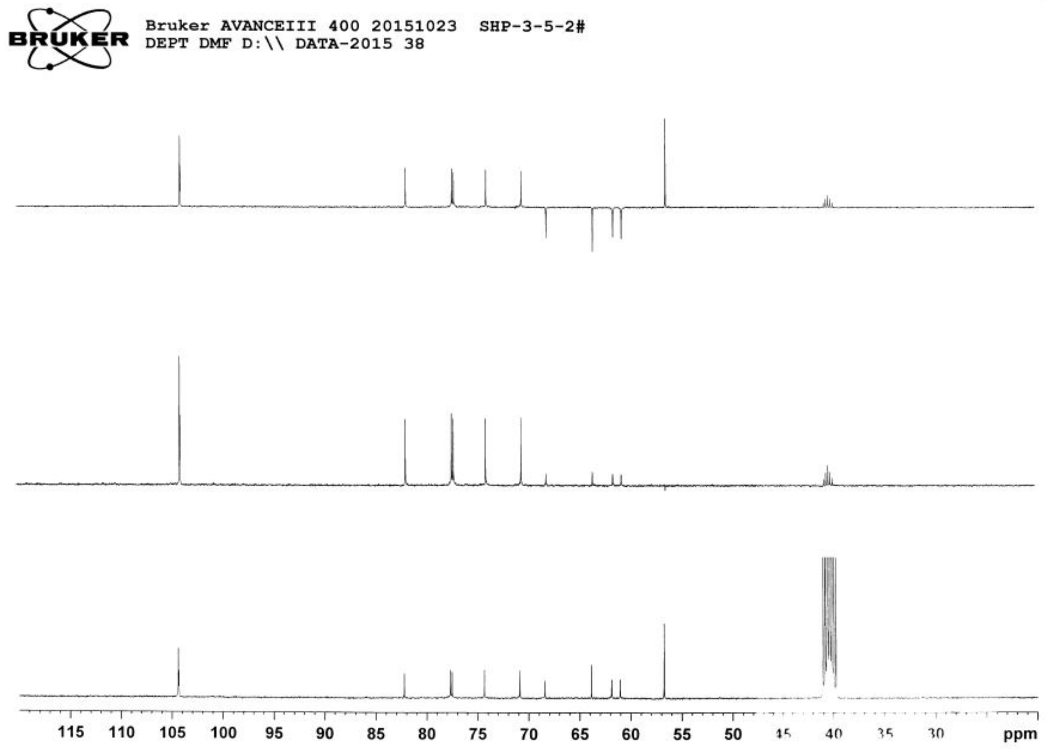

Figure S14. The DEPT Spectrum of compound 2

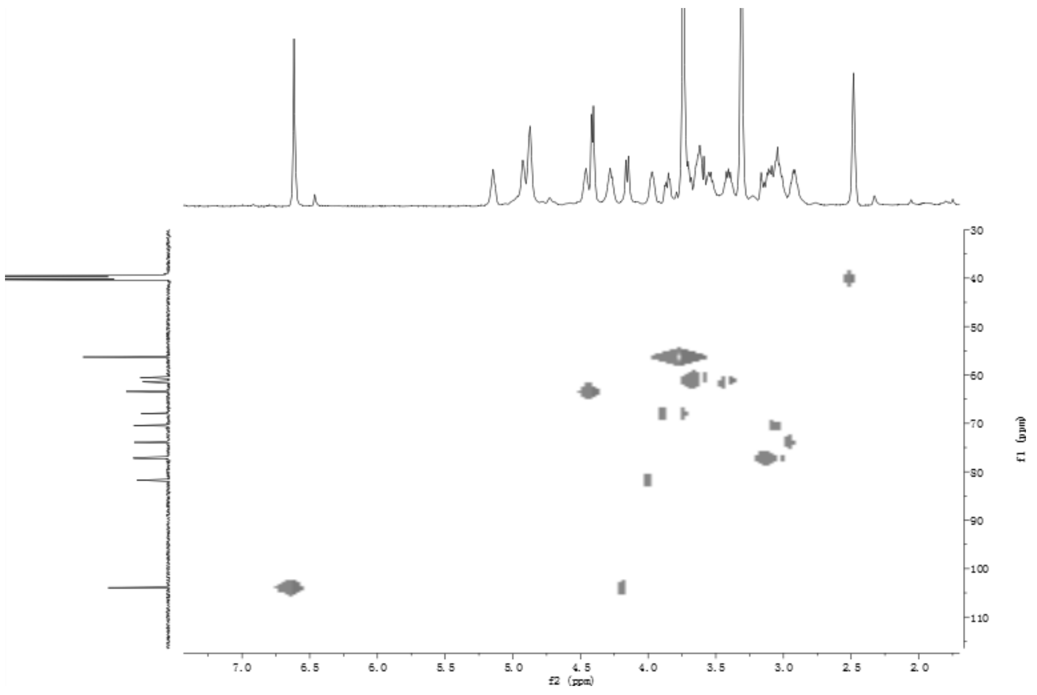

Figure S15. The HSQC Spectrum of compound 2

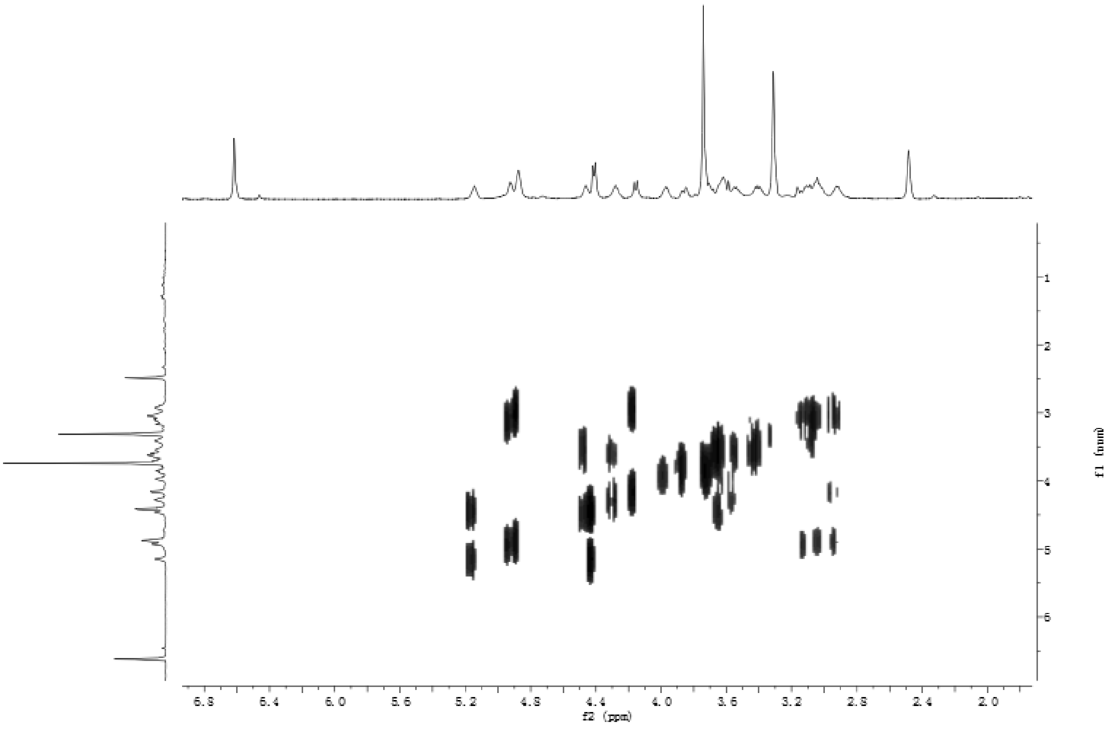

Figure S16. The ^{1}H, ^{1}H-COSY Spectrum of compound 2

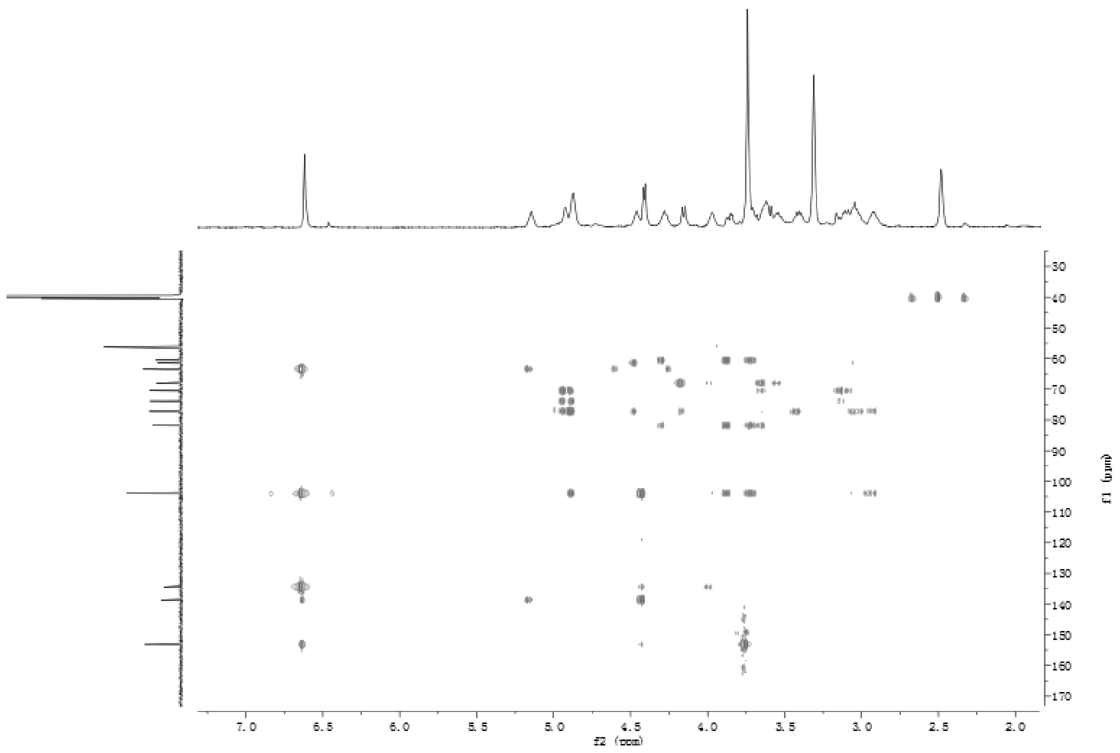

Figure S17. The HMBC Spectrum of compound 2

MS Formula Results: + Scan (3.831 min) Sub (2016031802.d)

m/z	Ion	Formula	Abundance
443.1532	(M+Na)+	C18 H28 Na O11	355264.8

Best	Formula (M)	Ion Formula	Score	Cross Sco	Mass	Calc Mass	Calc m/z	Diff (ppm)	Abs Diff (ppm)	Mass Match	Abund Match	Spacing Match	DBE
✔	C18 H28 O11	C18 H28 Na O11	99.89		420.164	420.1632	443.1524	-1.95	1.95	99.89	99.89	99.9	5
	C31 H20 N2	C31 H20 N2 Na	94.87		420.164	420.1626	443.1519	-3.17	3.17	99.7	82.71	99.81	23

Figure S18. The HRESIMS Spectrum of compound 2

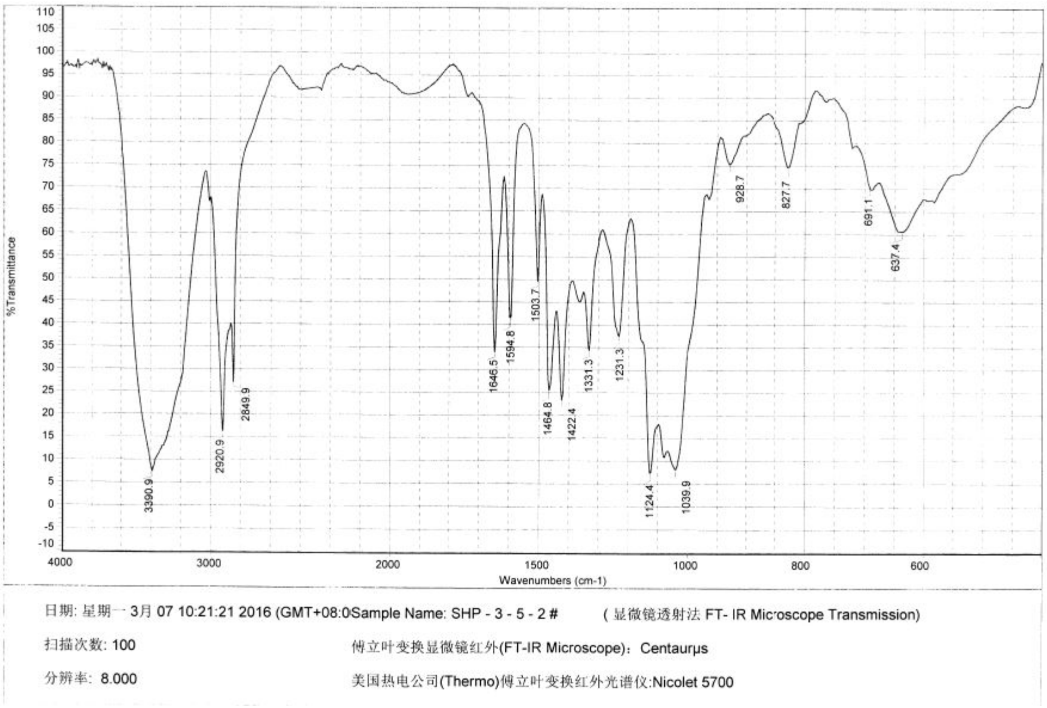

Figure S19. The IR Spectrum of compound 2

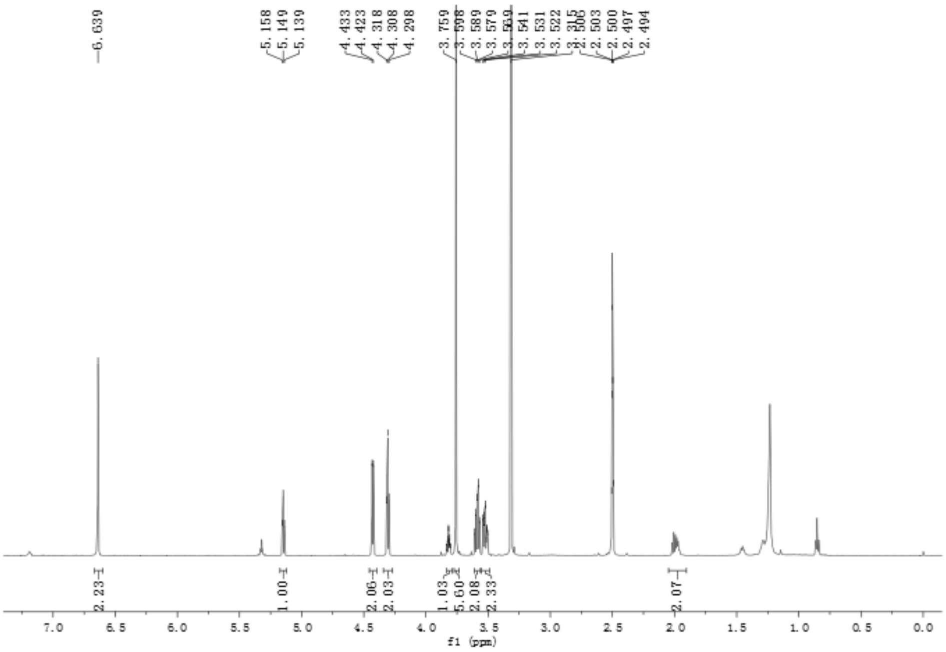

Figure S20. The ^{1}H NMR Spectrum of compound 2a（600 MHz，DMSO-*d*6）

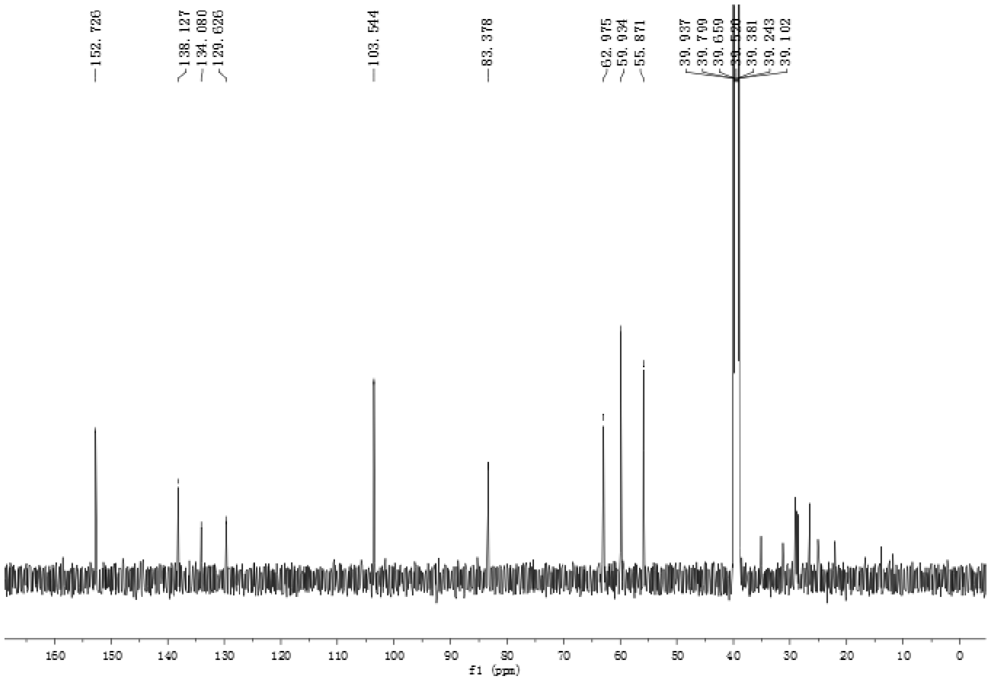

Figure S21. The ^{13}C NMR Spectrum of compound 2a（150 MHz，DMSO-*d*$_6$）

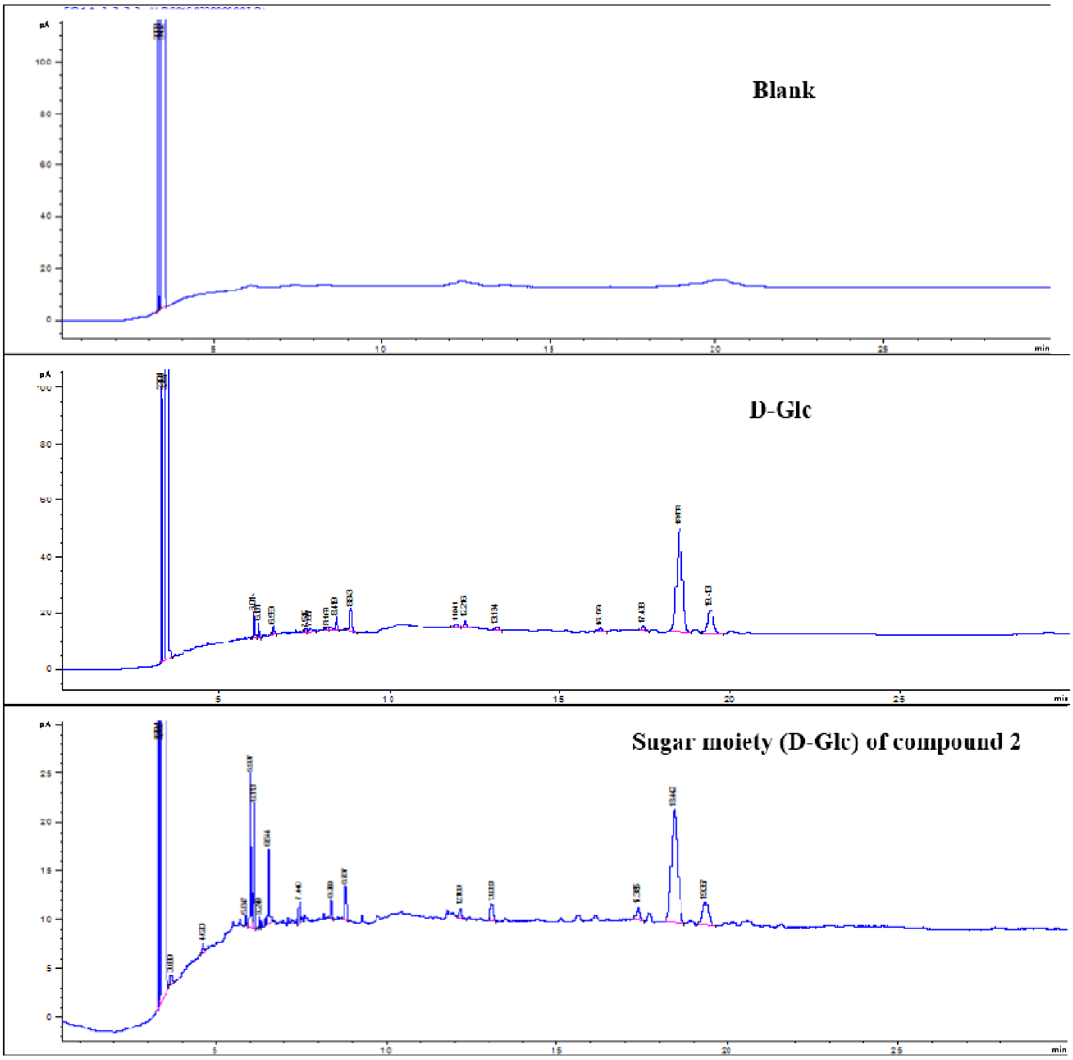

Figure S22. GC chromatogram of sugar motiety silylationed after acid hydrolysis of 2

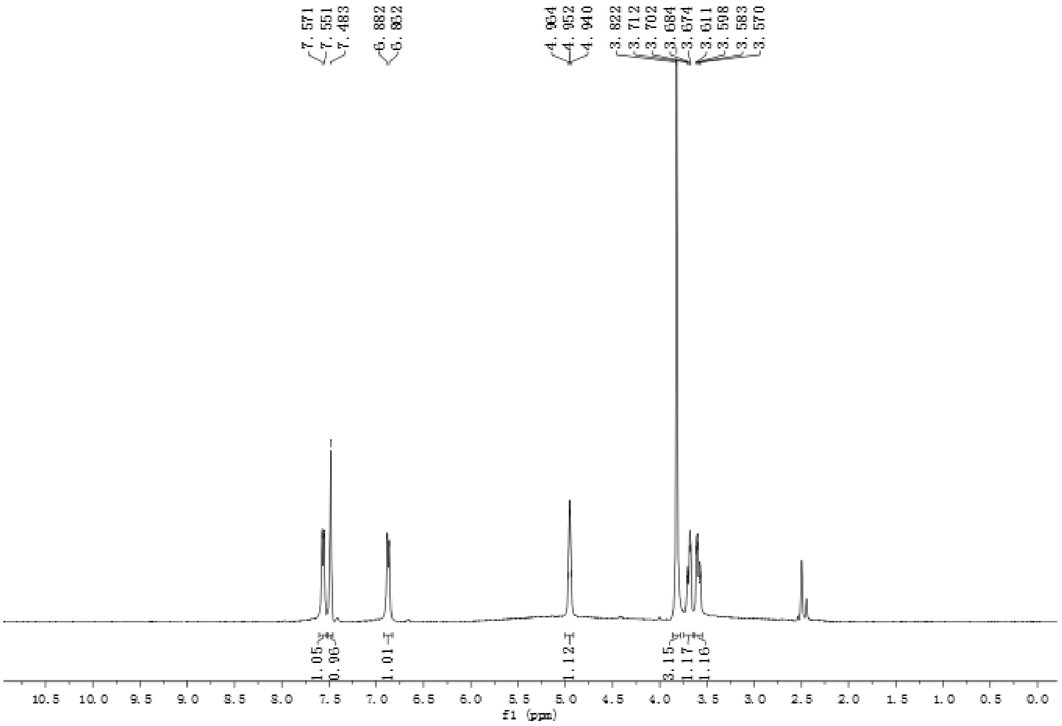

Figure S23. The ^{1}H NMR Spectrum of compound 3（400 MHz，DMSO-*d*₆）

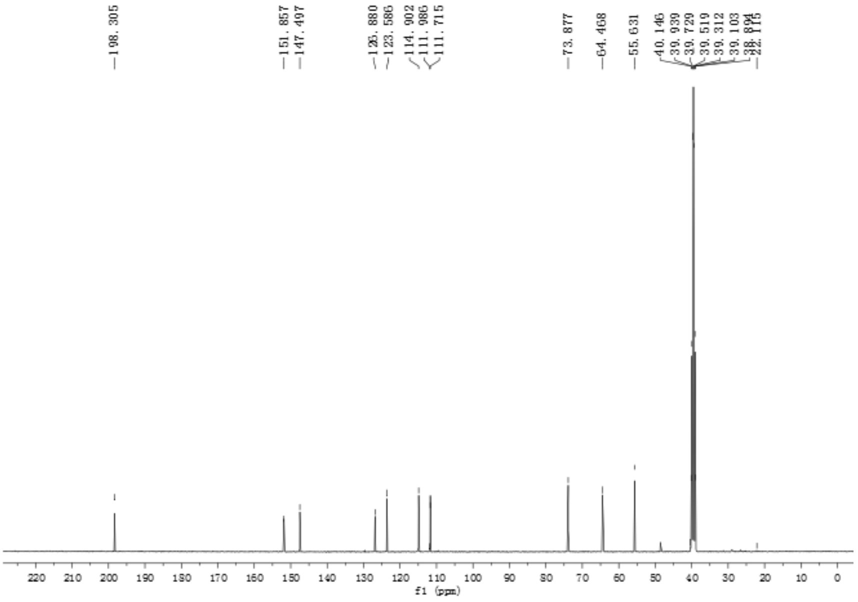

Figure S24. The ^{13}C NMR Spectrum of compound 3（100 MHz，DMSO-*d*$_6$）

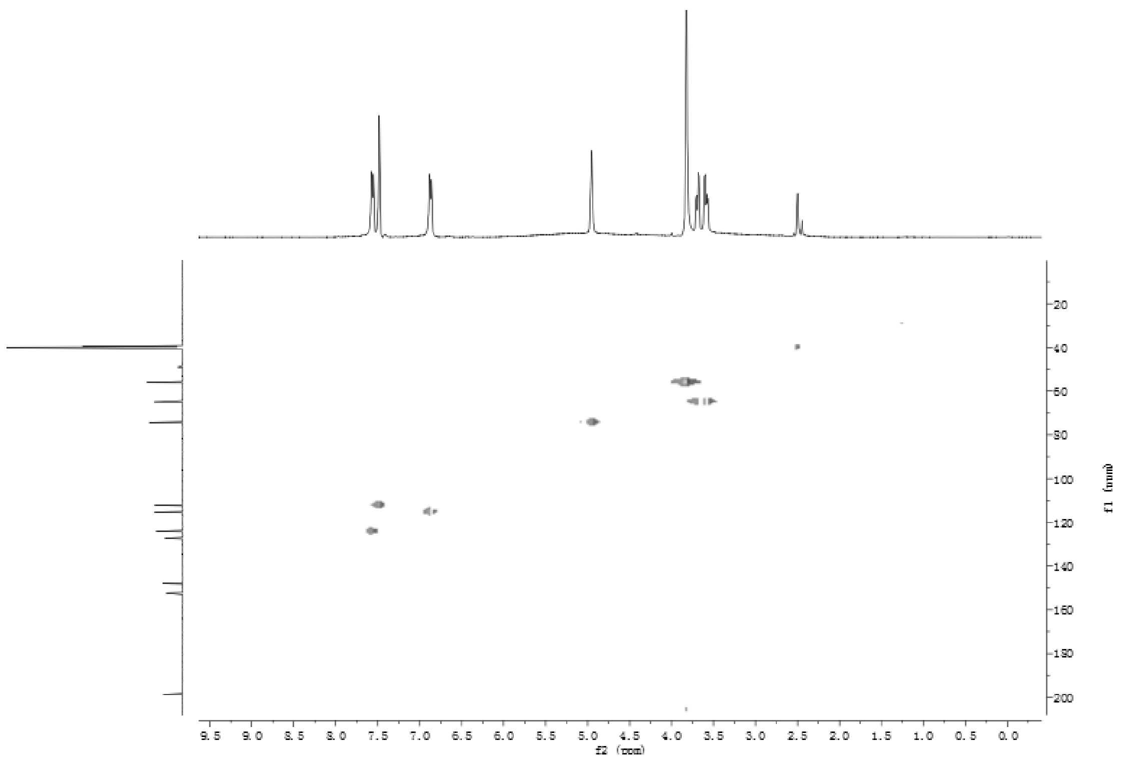

Figure S25. The HSQC Spectrum of compound 3

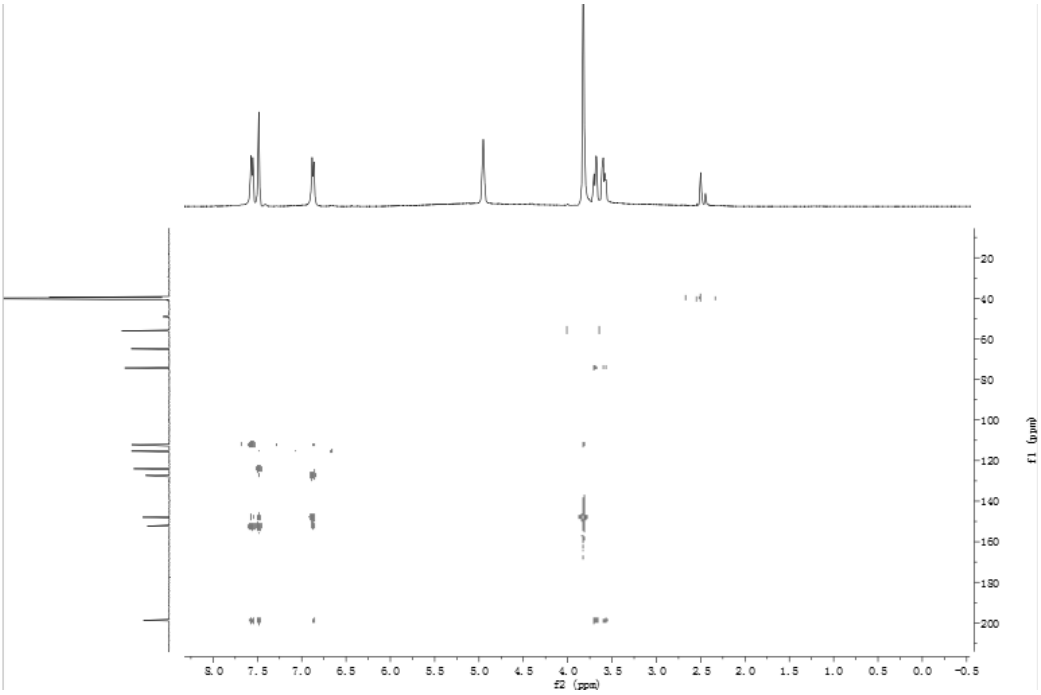

Figure S26. The HMBC Spectrum of compound 3

MS Formula Results: + Scan (1.789 min) Sub (2016031803.d)

m/z	Ion	Formula	Abundance
235.0573	(M+Na)+	C10 H12 Na O5	76301.2

Best	Formula (M)	Ion Formula	Score	Cross Sco	Mass	Calc Mass	Calc m/z	Diff (ppm)	Abs Diff (ppm)	Mass Match	Abund Match	Spacing Match	DBE
✔	C10 H12 O5	C10 H12 Na O5	99.92		212.0681	212.0685	235.0577	1.83	1.83	99.92	99.88	99.95	5

Figure S27. The HRESIMS Spectrum of compound 3

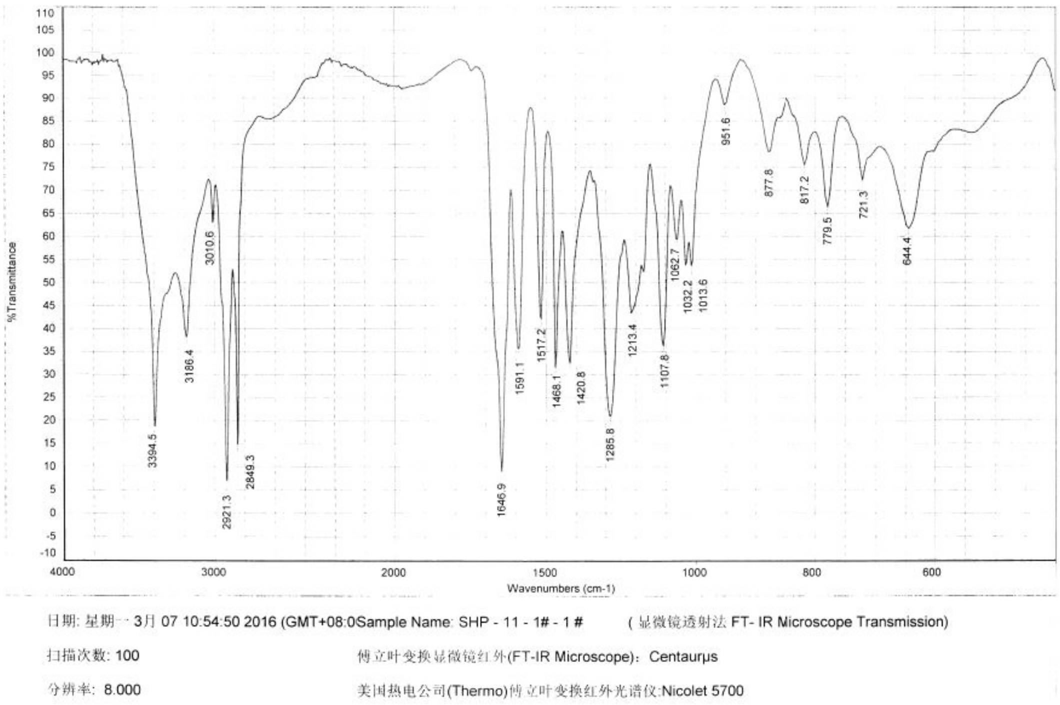

Figure S28. The IR Spectrum of compound 3a

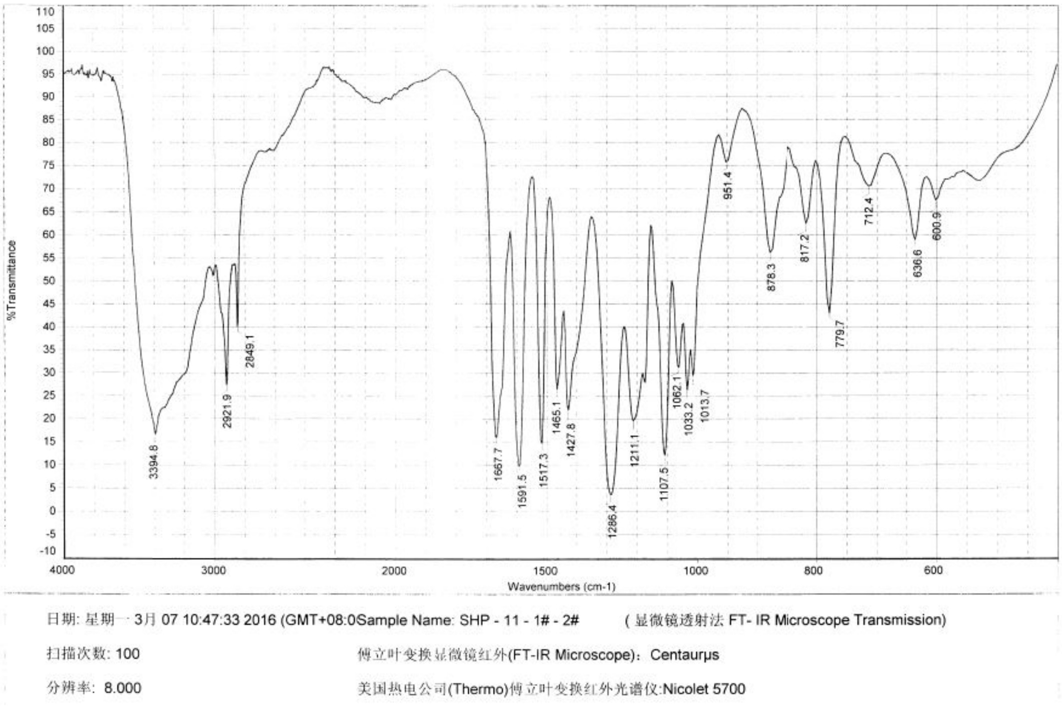

Figure S29. The IR Spectrum of compound 3b

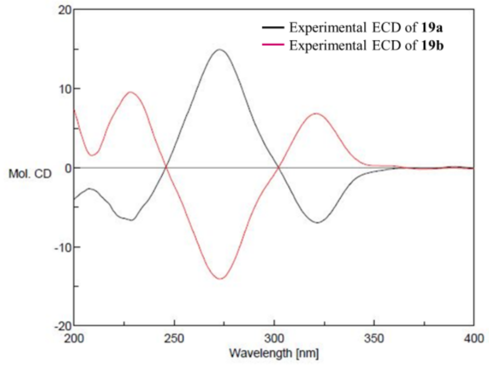

Figure S30. The experimental CD Spectrum of compound 3a and 3b in MeOH

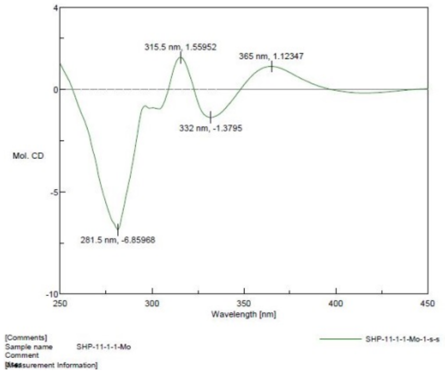

Figure S31. The $Mo_2(OAc)_4$ Induced CD Spectrum of compound 3a

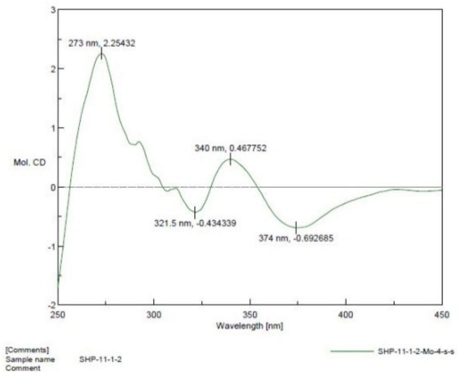

Figure S32. The $Mo_2(OAc)_4$ Induced CD Spectrum of compound 3b

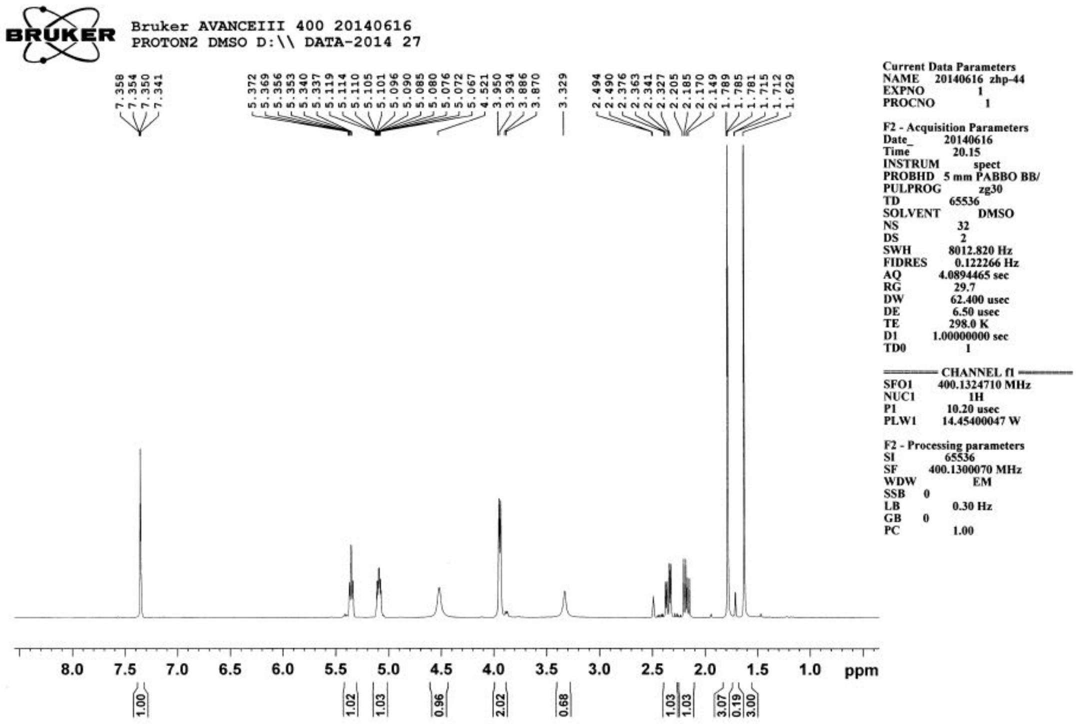

Figure S33. The ^{1}H NMR Spectrum of compound 4（600 MHz，DMSO-d_6）

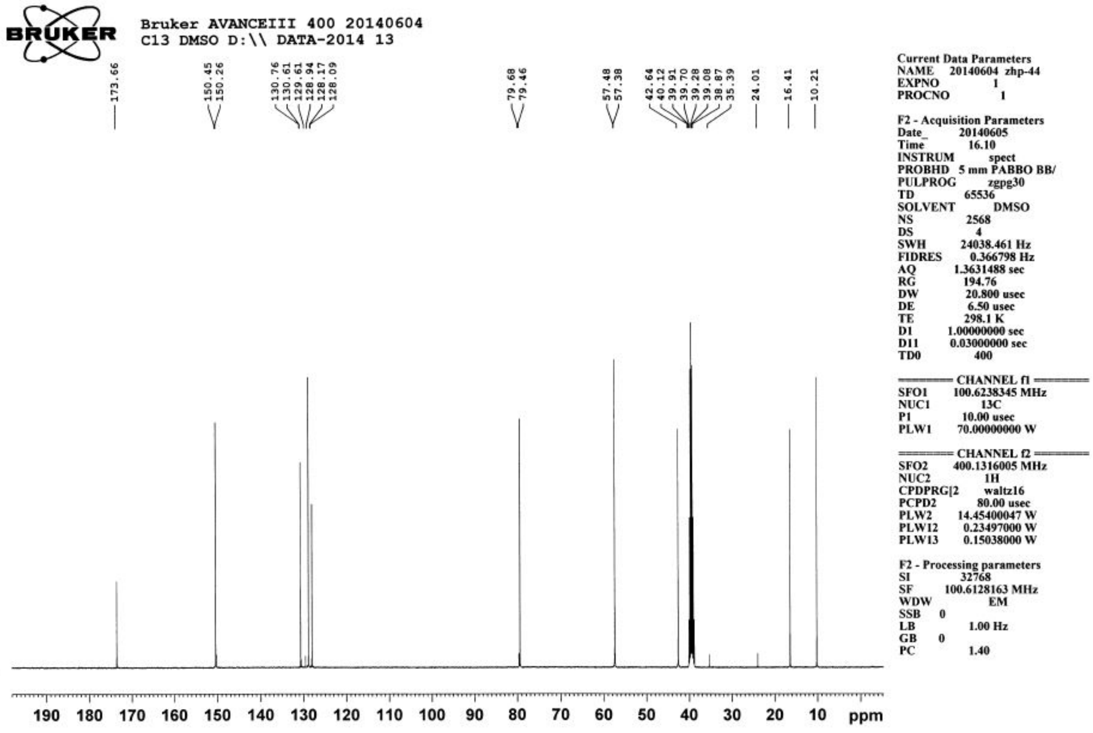

Figure S34. The ^{13}C NMR Spectrum of compound 4（150 MHz，DMSO-d_6）

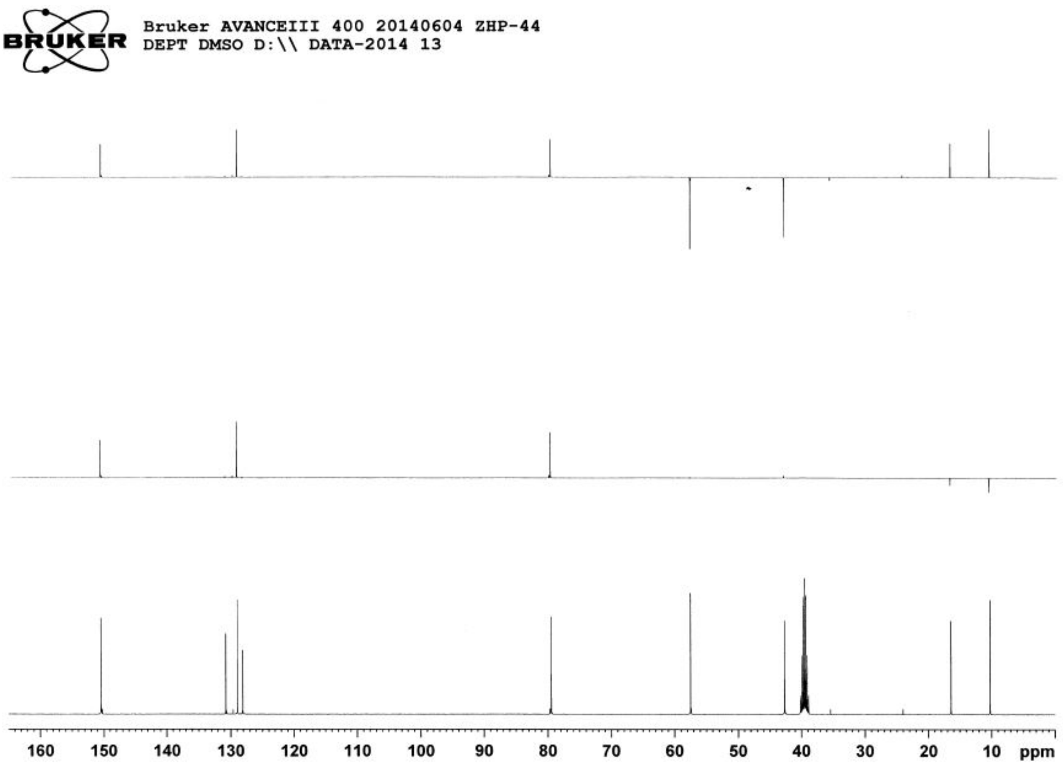

Figure S35. The DEPT Spectrum of compound 4

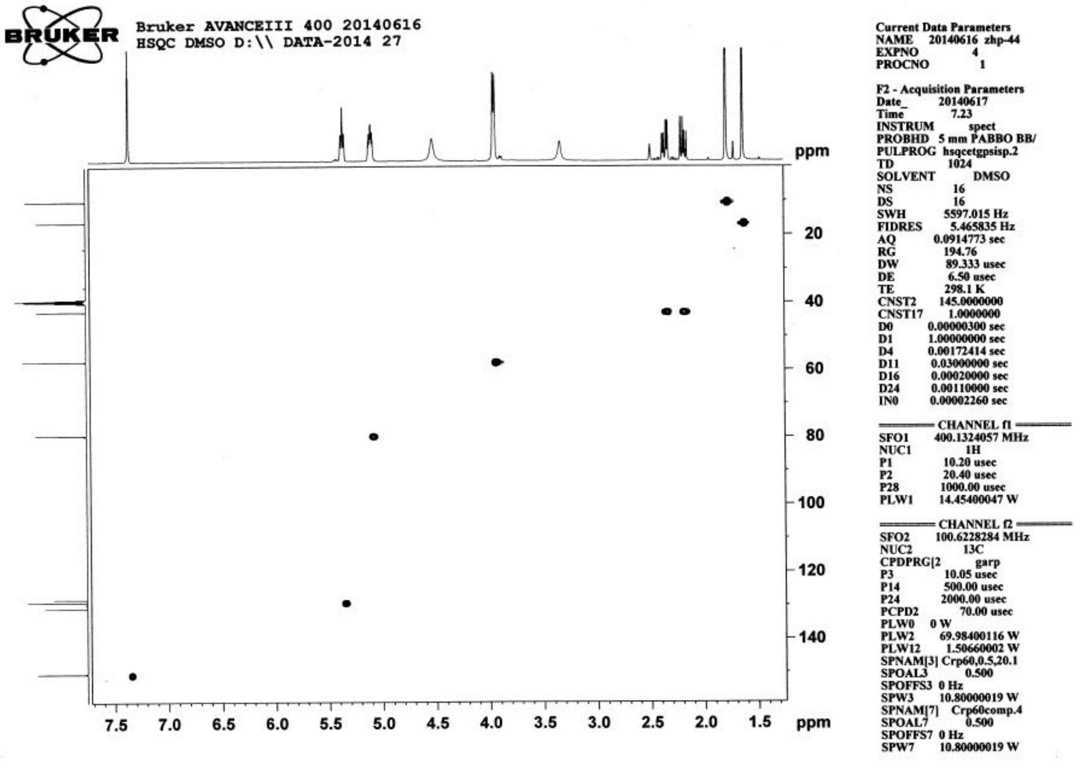

Figure S36. The HSQC Spectrum of compound 4

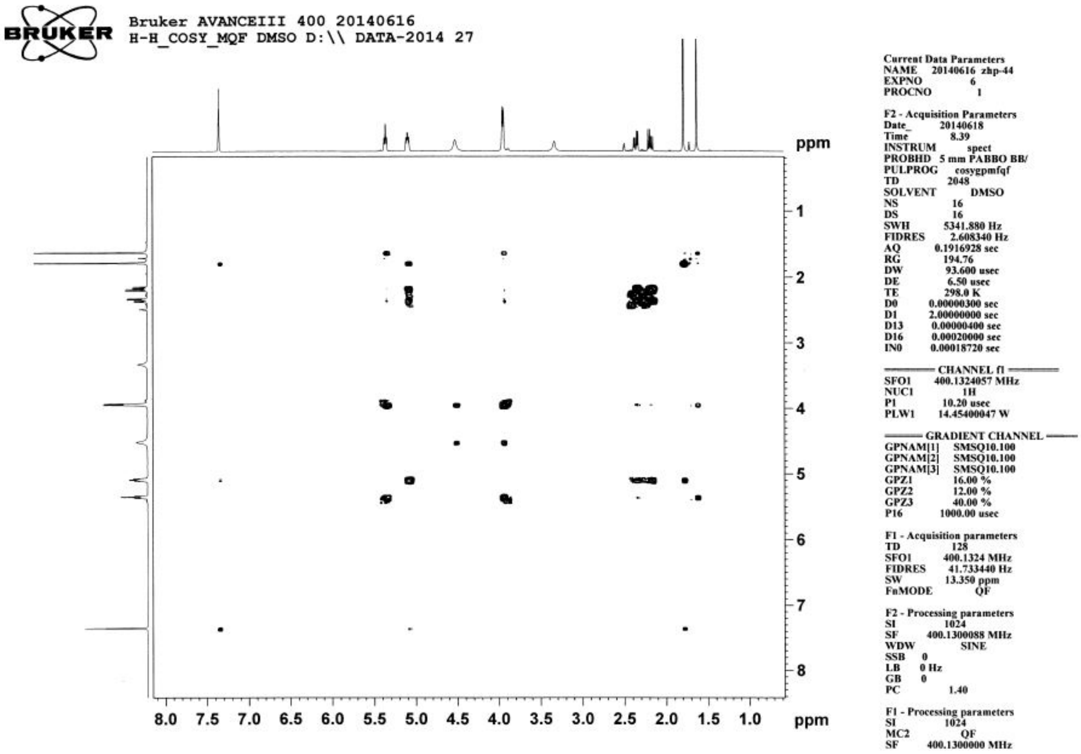

Figure S37. The ^1H, ^1H-COSY Spectrum of compound 4

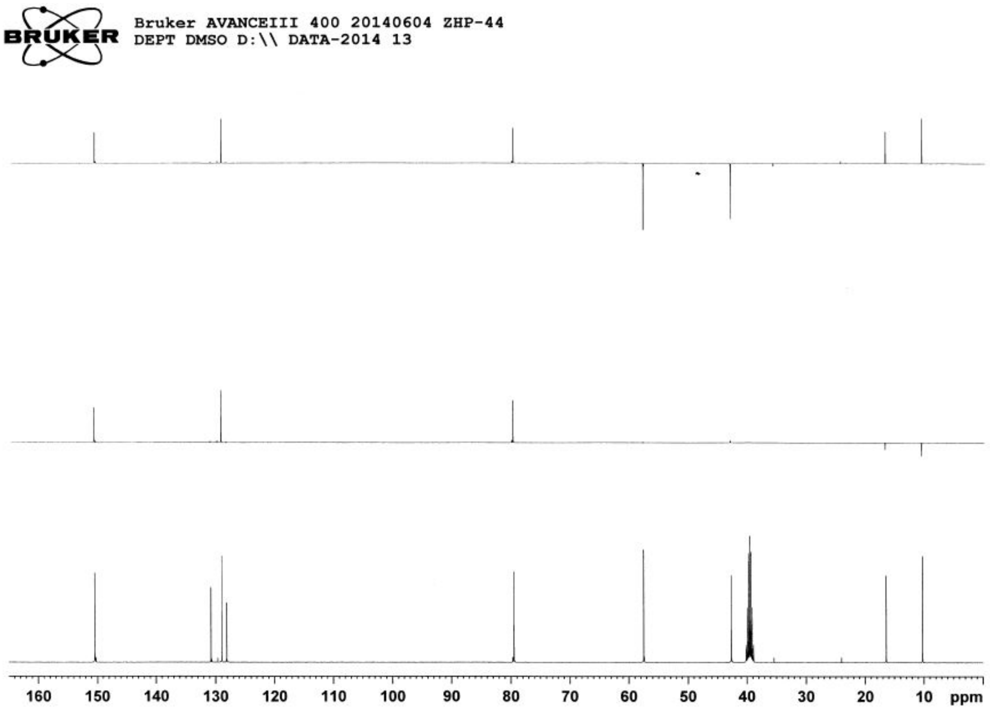

Figure S38. The HMBC Spectrum of compound 4

MS Formula Results: + Scan (4.334 min) Sub (2014061603.d)

m/z	Ion	Formula	Abundance
205.0835	(M+Na)+	C10 H14 Na O3	1367869.5

Best	Formula (M)	Ion Formula	Score	Cross Sco	Mass	Calc Mass	Calc m/z	Diff (ppm)	Abs Diff (ppm)	Mass Match	Abund Match	Spacing Match	DBE
☑	C10 H14 O3	C10 H14 Na O3	99.99		182.0942	182.0943	205.0835	0.33	0.33	100	99.99	99.98	4

Figure S39. The HRESIMS Spectrum of compound 4

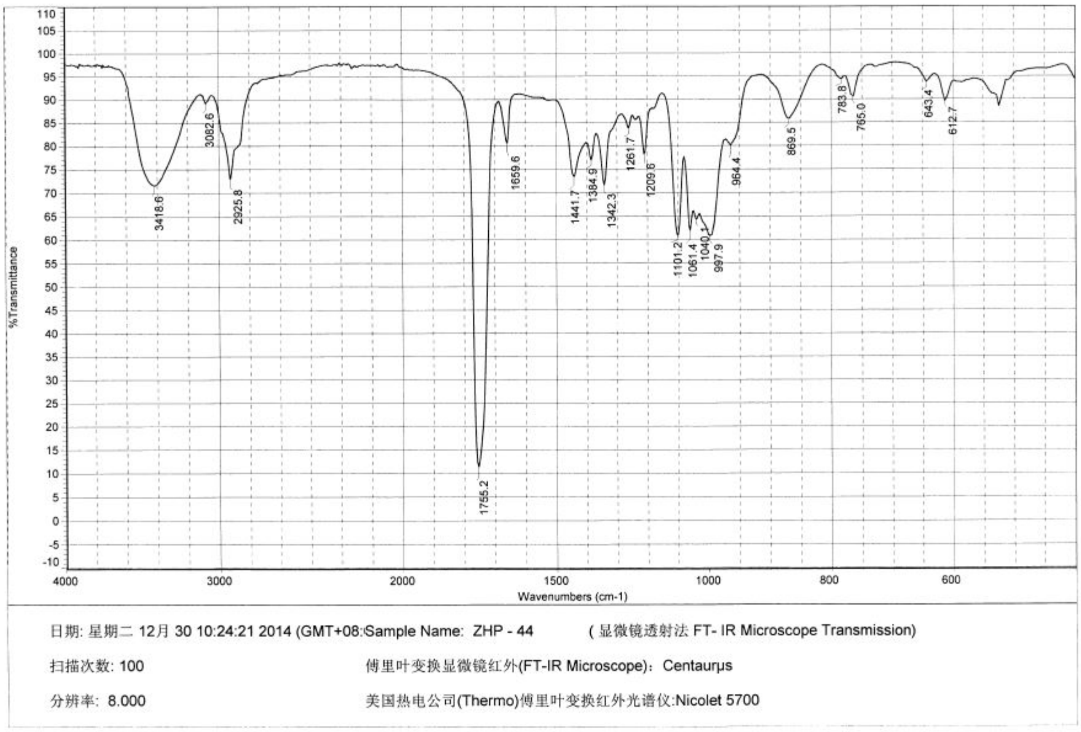

Figure S40. The IR Spectrum of compound 4

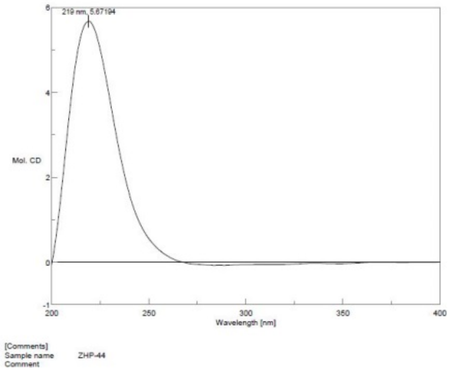

Figure S41. The CD Spectrum of compound 4

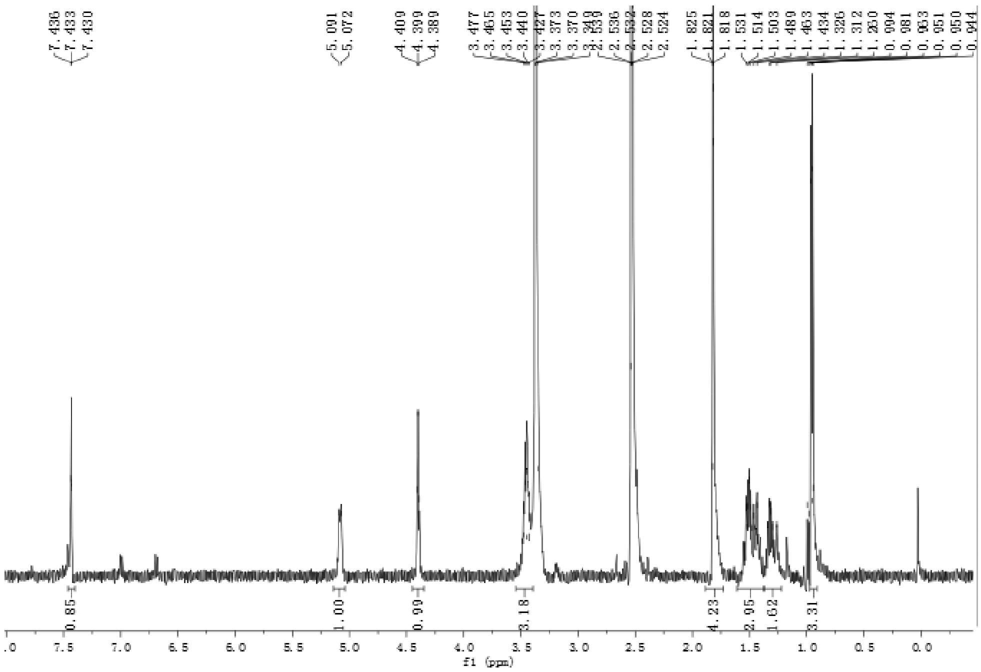

Figure S42. The ^{1}H NMR Spectrum of compound 5（600 MHz，DMSO-d_6）

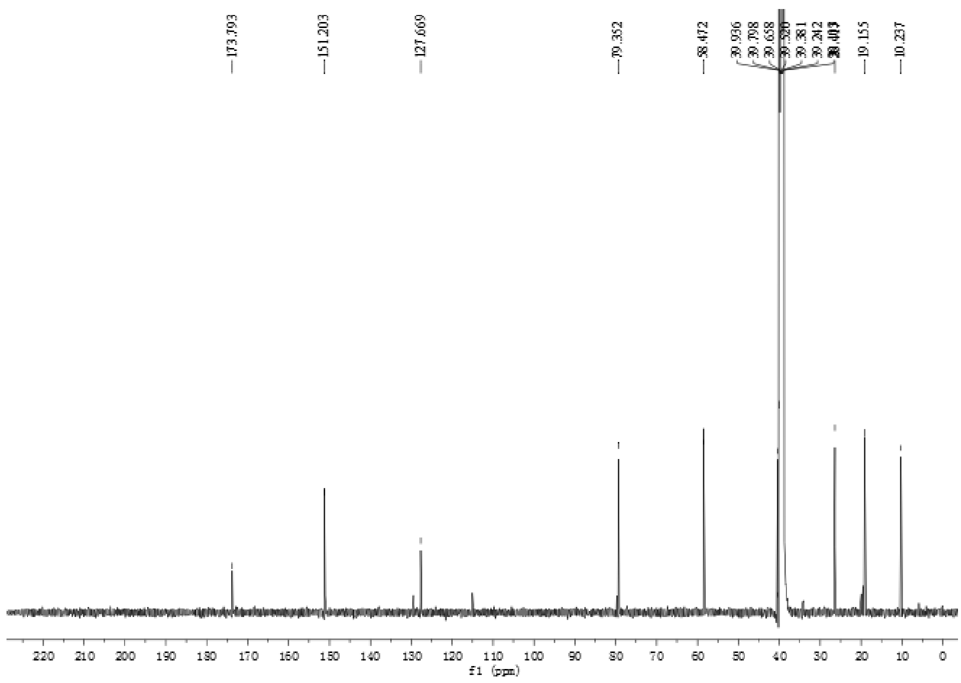

Figure S43. The ^{13}C NMR Spectrum of compound 5（150 MHz，DMSO-d_6）

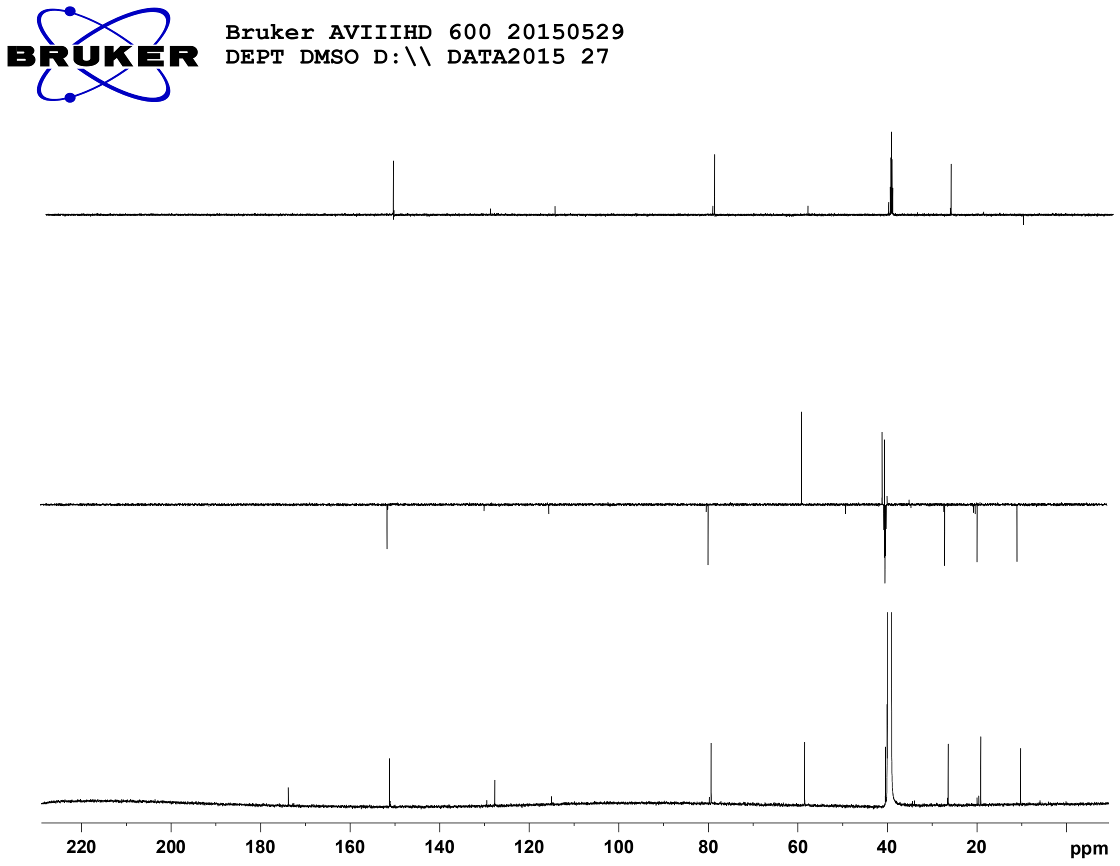

Figure S44. The DEPT Spectrum of compound 5

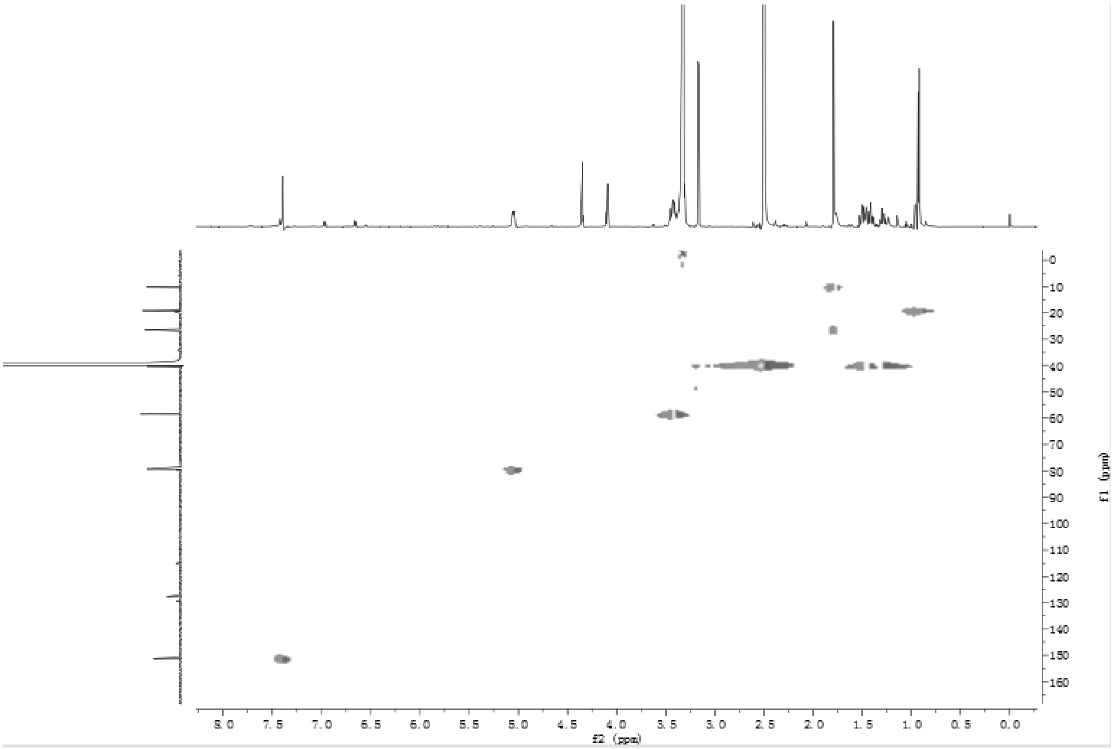

Figure S45. The HSQC Spectrum of compound 5

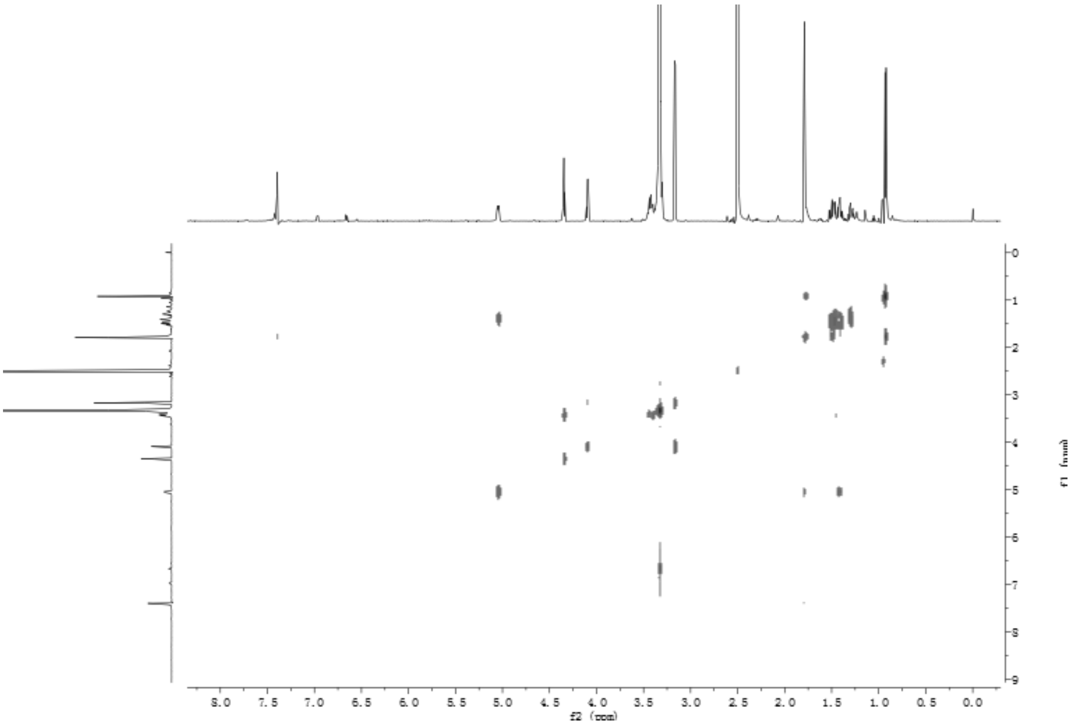

Figure S46. The ^{1}H, ^{1}H-COSY Spectrum of compound 5

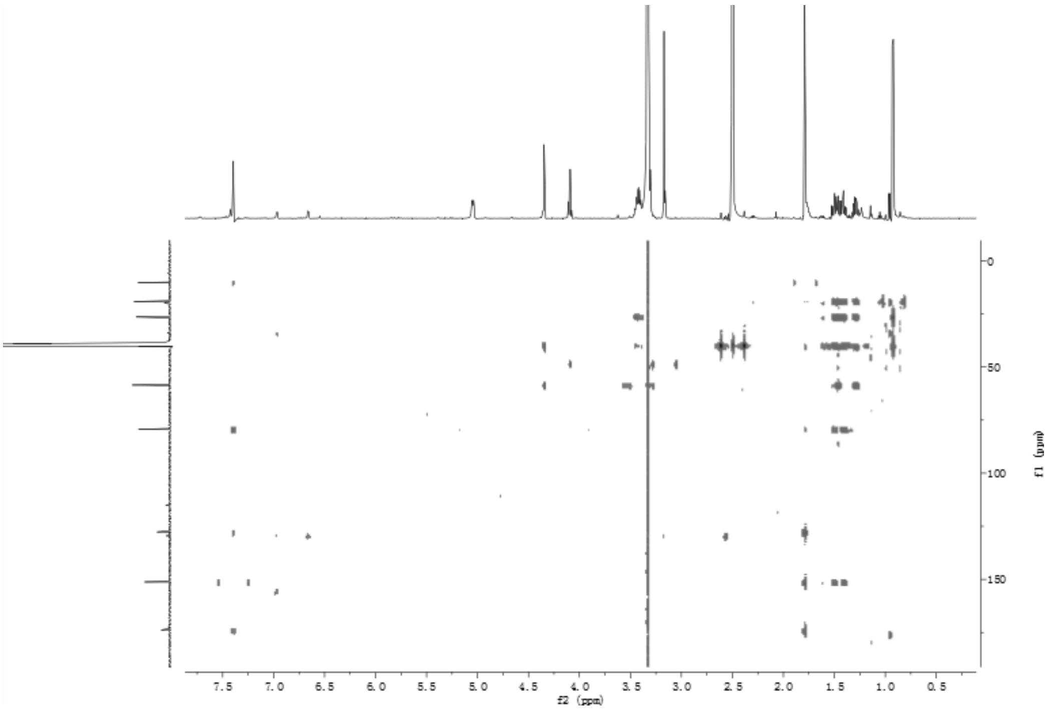

Figure S47. The HMBC Spectrum of compound 5

MS Formula Results: + Scan (4.882 min) Sub (2016031804.d)

m/z	Ion	Formula	Abundance
207.0993	(M+Na)+	C10 H16 Na O3	89198.8

Best	Formula (M)	Ion Formula	Score	Cross Sco	Mass	Calc Mass	Calc m/z	Diff (ppm)	Abs Diff (ppm)	Mass Match	Abund Match	Spacing Match	DBE
✔	C10 H16 O3	C10 H16 Na O3	97.42		184.1101	184.1099	207.0992	-0.64	0.64	99.99	99.65	89.59	3

Figure S48. The HRESIMS Spectrum of compound 5

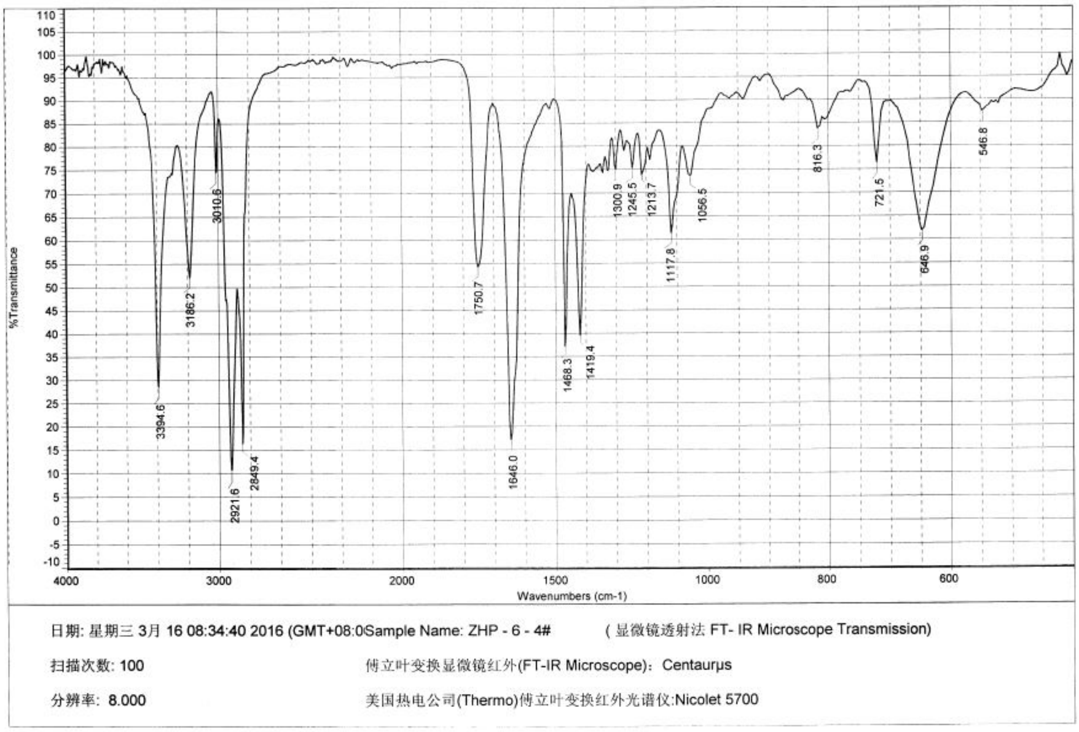

Figure S49. IR Spectrum of compound 5

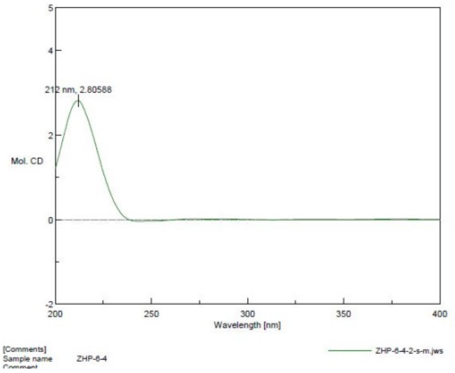

Figure S50. CD Spectrum of compound 5
